# IGF2BP2 Deficiency in Macrophages Impairs Migration, Reprograms Metabolism, and Limits Tumor Progression

**DOI:** 10.7150/ijbs.122142

**Published:** 2026-02-18

**Authors:** Hanna S. Schymik, Selina Wrublewsky, Marcus Höring, Gerhard Liebisch, Simon Both, Gilles Gasparoni, Caroline Bickelmann, Hanah Robertson, Charlotte Dahlem, Jörn Walter, Volkhard Helms, Matthias W. Laschke, Emmanuel Ampofo, Jessica Hoppstädter, Alexandra K. Kiemer

**Affiliations:** 1 Department of Pharmacy, Pharmaceutical Biology, Saarland University, Saarbrücken, Germany.; 2 Institute for Clinical & Experimental Surgery, Saarland University, Homburg, Germany.; 3 Institute of Clinical Chemistry and Laboratory Medicine, University Hospital Regensburg, Regensburg, Germany.; 4 Department of Genetics/Epigenetics, Saarland University, Saarbrücken, Germany.; 5 Center for Bioinformatics, Saarland University, Saarbrücken. Germany; 6 Center for Gender-Specific Biology and Medicine (CGBM), Saarland University, Homburg, Germany.; 7 PharmaScienceHub (PSH), Saarland University, Saarbrücken, Germany.

## Abstract

While insulin-like growth factor 2 mRNA-binding protein 2 (IGF2BP2) has been extensively studied in tumor cells, its role in immune cells within the tumor microenvironment, particularly in macrophages, remains largely unknown. Here, we reveal a critical function of IGF2BP2 in macrophages, demonstrating that myeloid-specific deletion of IGF2BP2 profoundly alters macrophage metabolism and polarization, and markedly impairs tumor progression.

Bulk RNA sequencing of IGF2BP2 knockout (KO) macrophages revealed significant alterations in gene expression profiles, particularly impacting pathways associated with glycolysis, mitochondrial function, cell motility, and cell migration. Functional assays confirmed increased glycolytic activity and a concomitant reduction in maximal respiration and reserve respiratory capacity, indicating a metabolic shift towards glycolysis. Furthermore, IGF2BP2 deficiency impaired tumor-associated macrophage (TAM)-like polarization *in vitro*, as evidenced by decreased expression of TAM markers, such as *Mrc1*, *Mmp2*, and *Il10*. Lipidomic profiling revealed distinct lipid signatures in IGF2BP2 KO TAM-like macrophages, including alterations in triglycerides and cardiolipins, crucial for mitochondrial integrity.

*In vivo*, deletion of IGF2BP2 specifically in the myeloid lineage was sufficient to reduce tumor growth in a subcutaneous Lewis lung carcinoma model, accompanied by decreased TAM infiltration and a shift towards a pro-inflammatory macrophage phenotype. Additionally, IGF2BP2-deficient macrophages showed impaired migratory capacity both *in vitro* and *in vivo*. These findings underscore the critical role of IGF2BP2 in controlling macrophage metabolism, polarization, and tumor-supporting functions within the tumor microenvironment, and identify myeloid IGF2BP2 as a potential therapeutic target in cancer.

## Introduction

Macrophages are essential components of the immune system, playing a central role in immune defense, inflammation, and tissue homeostasis. Their ability to polarize into distinct functional states, including the classically activated M1 and alternatively activated M2 phenotypes, enables them to adapt to diverse physiological and pathological conditions 1,2. In the tumor microenvironment, macrophages often adopt a tumor-associated phenotype (TAM), predominantly immunosuppressive and pro-tumorigenic. These cells contribute to tumor progression by promoting angiogenesis, immune evasion, and extracellular matrix remodeling 3,4. Despite considerable progress in understanding macrophage biology, the molecular mechanisms governing their polarization and metabolic reprogramming remain incomplete.

The insulin-like growth factor 2 mRNA-binding protein 2 (IGF2BP2/IMP2) is an RNA-binding protein that regulates gene expression post-transcriptionally, primarily through recognition of N6-methyladenosine (m6A) modifications 5. IGF2BP2 is implicated in diverse cellular processes, including metabolism, differentiation, and oncogenesis, with dysregulated expression linked to poor prognosis in various cancers, such as colorectal, lung, bladder, and hepatocellular carcinoma 6-8. Beyond its role in cancer, IGF2BP2 is a key regulator of metabolic pathways, influencing glucose tolerance, insulin sensitivity, and fatty acid oxidation, and is associated with metabolic disorders, such as diabetes and obesity 9.

Understanding the molecular mechanisms that govern macrophage function in cancer is crucial, particularly for aggressive cancers with high mortality rates. Among these, lung cancer stands out as both the most frequently diagnosed malignancy and the leading cause of cancer-related deaths worldwide. In 2022, lung cancer accounted for approximately 2.48 million new cases, representing 11.6% of all cancer diagnoses globally 10,11. Notably, increased expression of IGF2BP2 has been consistently observed in lung cancer tissues, especially in cases resistant to conventional treatments. This elevated IGF2BP2 expression is associated with tumor progression and therapy resistance, highlighting its potential as a critical factor in lung cancer pathogenesis 12,13. Although targeted therapies and immune checkpoint inhibitors have advanced treatment options, the majority of lung cancer patients still face poor outcomes due to resistance mechanisms, often driven by TAM infiltration 14,15.

Emerging evidence also indicates that IGF2BP2 is involved in inflammatory regulation, influencing immune responses in rheumatoid arthritis, periodontitis, ulcerative colitis, and steatohepatitis 16-19. This regulation is primarily mediated through the stabilization of mRNAs encoding inflammatory mediators. However, its role in macrophage polarization—particularly in TAMs—remains poorly understood. Given the critical function of TAMs in linking chronic inflammation to cancer progression, elucidating the influence of IGF2BP2 on macrophage behavior could provide novel insights into tumor immunology and potential therapeutic targets. Notably, our previous work demonstrated that IGF2BP2 is highly expressed in M2 macrophages and chronic inflammatory diseases 20, further suggesting its potential involvement in shaping the tumor microenvironment.

Importantly, while IGF2BP2 is known to promote tumor progression in various cancers through tumor-intrinsic mechanisms, its function within macrophages, especially tumor-associated macrophages (TAMs), has not been systematically addressed. Our study is the first to demonstrate that deletion of IGF2BP2 in myeloid cells, rather than in tumor cells, results in markedly reduced tumor growth and reveals a previously unrecognized role of IGF2BP2 in macrophage-mediated tumor support.

## Materials & Methods

### Materials

All materials and reagents used in this study were obtained from commercial suppliers. RPMI-1640 cell culture medium (#R0833) and DMEM (high Glucose) cell culture medium (#D6546-0500) were purchased from Sigma-Aldrich (St. Louis, USA), along with L-glutamine (#G7513), penicillin/streptomycin (#P4333), Accutase (#A6964), Trypsin-EDTA (#T3924), and Antimycin A (#A8674, sourced from Streptomyces sp.). Filtrated bovine serum (FBS, #P040-37500) was obtained from PAN Biotech (Aidenbach, Germany). Blasticidin S hydrochloride (#R21001) was acquired from Thermo Fisher Scientific (Waltham, USA). Other chemicals were purchased either from Sigma-Aldrich (St. Louis, USA) or Carl Roth (Karlsruhe, Germany) unless stated otherwise.

### Mice

Mice were housed under a 12-hour light/dark cycle and* ad libitum* access to food and water. Myeloid-specific IGF2BP2 knockout (IGF2BP2 ΜΦ-KO) mice were generated by crossing B6.Cg-Igf2bp2tm1.1Thor/J mice (The Jackson Laboratory, Bar Harbor, USA), which carry loxP sites flanking exons 4 and 5 of the *Igf2bp2* gene, with 29P2-Lyz2tm1(cre)Ifo/J mice (The Jackson Laboratory Bar Harbor, USA). Control wild-type (WT) mice were littermates that did not carry floxed alleles and either expressed or lacked Cre recombinase. IGF2BP2 ΜΦ-KO mice were validated by assessing protein and mRNA levels. Sex- and age-matched littermates were used for all experiments, except for tumor studies, which were conducted exclusively in female mice. All animal procedures were approved by the local animal welfare committee (AZ 2.4.1.1; AZ06-2020; AZ04-2023; Saarland State Office for Social Affairs, Health, and Consumer Protection).

### Cell Culture Conditions

Cells were cultured at 37°C in a humidified 5% CO₂ atmosphere in RPMI-1640 medium supplemented with 10% FBS, 2 mM L-glutamine, and 100 units/ml penicillin G, 100 μg/ml streptomycin, unless stated otherwise.

### Isolation and Cultivation of Bone Marrow-Derived Macrophages

Bone marrow cells were isolated from three- to four-month-old WT or IGF2BP2 ΜΦ-KO mice. Cells were flushed from femurs and tibias using a standard medium (RPMI 1640 supplemented with 10% fetal bovine serum [FBS], 100 U/ml penicillin G, 100 μg/ml streptomycin, and 2 mM glutamine). After centrifugation at 200×*g* for 10 minutes, erythrocytes were lysed by incubation in hypotonic buffer (155 mM NH₄Cl, 10 mM KHCO₃, and 1 mM Na₂EDTA) for 3 minutes at 37°C. The remaining cells were resuspended in a standard medium supplemented with macrophage colony-stimulating factor (M-CSF, 50 ng/ml, #130-101-704, Miltenyi Biotec Bergisch Gladbach, Germany) and transferred to a 75 cm² culture flask overnight for initial attachment. Non-adherent cells were collected the following day and cultured in a 150 cm² culture flask for 5-6 days in an M-CSF-containing medium to promote differentiation into bone marrow-derived macrophages (BMMs), as described before 21. Differentiated cells were detached using Accutase, resuspended in a standard medium supplemented with 50 ng/ml M-CSF, and seeded as indicated. To polarize or activate BMMs, the differentiation medium was supplemented with 20 ng/ml recombinant interferon (IFN)-γ (#87389.20, Biomol, Hamburg, Germany) and 100 ng/ml lipopolysaccharide (LPS) (from E. coli K12, #tlrl-peklps, Invivogen, Toulouse, France) for M1 polarization, either 20 ng/ml interleukin-4 (IL-4) (#130-093-921, Miltenyi Biotech, Bergisch Gladbach, Germany) for M2 polarization, or left without further supplementation for M0 macrophages. TAM-like macrophages were generated with tumor-conditioned medium (TCM) supplemented with 50 ng/ml M-CSF. TCM was generated by seeding 0.5-1 × 10^6^ murine Lewis lung carcinoma (LLC1, CRL-1642, ATCC, Manassas, USA) cells into a T75 culture flask and growing until confluency for three days. Subsequently, the supernatant was discarded, and a standard growth medium was added to the cells. After 48 hours, the medium was sterile-filtered (0.22 µm) to remove cell debris. For macrophage polarization, TCM was used directly without further dilution (100% v/v) and supplemented with 50 ng/mL M-CSF. IL-4 and M-CSF were prepared with sterile water for cell biology (#95289, Sigma-Aldrich, St. Louis, USA, ≤ 0.005 EU/mL endotoxins).

### RNA Sequencing (RNA-Seq)

Next-generation sequencing (NGS) was performed to analyze the transcriptome of BMMs from WT and IGF2BP2 ΜΦ-KO mice, as previously described 22. 5 × 10^5^ BMMs were seeded in a 12-well plate and treated with 20 ng/ml IL-4 for 8 hours the following day or left untreated to generate M0 macrophages. Total RNA was isolated using the High Pure RNA Isolation Kit (#11828665001, Roche, Basel, Switzerland) according to the manufacturer's protocol. RNA quality was assessed using the 2100 Bioanalyzer (Agilent, Santa Clara, USA) with the RNA 6000 Nano Kit (#5067-1513, Agilent Technologies, Santa Clara, USA), and only RNA samples with an RNA integrity number (RIN) > 9 were used for further analysis. For library preparation, 500 ng of RNA was used. Poly(A) enrichment was performed using the NEBNext Poly(A) mRNA Magnetic Isolation Module (#E7490, New England Biolabs, Ipswich, USA) following the manufacturer's instructions. According to the manufacturer's protocol, cDNA libraries were generated using the NEBNext Ultra Directional RNA Library Prep Kit for Illumina (#E7420, New England Biolabs, Ipswich, USA). First- and second-strand cDNA synthesis was followed by adapter ligation and PCR amplification (12 cycles) to generate the final library. PCR products were purified using Agencourt AMPure XP beads (#A63881, Beckman Coulter, Brea, USA). Sequencing was performed in single-end mode (1 × 75 nt) using the NextSeq 500 (Illumina, San Diego, USA).

### RNA-Seq Data Processing and Analysis

Raw sequencing reads were demultiplexed and assessed for quality using FastQC v0.11.2. Reads were processed using the Grape-nf pipeline (v1.1.3) with Nextflow (v20.10.0) and aligned to the GRCm38/mm10 genome assembly. Differential expression analysis was performed using DESeq2 (v1.40.2) based on the read counts obtained after alignment. Principal component analysis (PCA) was conducted using transcripts-per-million (TPM) values for all annotated protein-coding genes. All statistical analyses were performed in R, as previously described 23. DESeq2 analysis identified differentially expressed genes (DEGs, *p* < 0.05) in IGF2BP2 ΜΦ-KO vs. WT macrophages under untreated and IL-4-treated conditions. TPM values of DEGs were then subjected to unsupervised k-means clustering using iDEP 1.12, independently for untreated and IL-4-treated cells. Pathway enrichment analysis was performed using Ingenuity Pathway Analysis (IPA), version 76765844 (QIAGEN, Venlo, Netherlands, June 2023), to identify significantly enriched canonical pathways.

### Seahorse Measurement

The Glycolytic and Mito Stress Tests (#103020-100, #103010-100, Agilent Technologies, Santa Clara, USA) were conducted using the Agilent Seahorse^®^ XF96 Analyzer and corresponding assay kits, following the manufacturer's protocols. Cells were seeded into Agilent Seahorse XF96 cell culture microplates (#103793-100, 5 × 10^4^ cells per well) and treated with IL-4 and LLC1 supernatant for 8 hours. The culture medium was replaced with Seahorse assay medium one hour prior to measurement.

During the Mito Stress Test, cells were treated with 1.5 µM carbonyl cyanide-4-(trifluoromethoxy)phenylhydrazone (FCCP) (Agilent Technologies, Santa Clara, USA). Other assay components were injected as recommended by the supplier. Oxygen consumption rate (OCR) and extracellular acidification rate (ECAR) were quantified using the Seahorse Wave software (Agilent Technologies, Santa Clara, USA). Data normalization was performed based on cell areas obtained from brightfield images and analyzed with Gen5 software (version 3.14) on a Cytation 1 imaging system (BioTek Instruments, Winooski, USA).

### Measuring Mitochondrial DNA Copy Number

Genomic DNA was extracted from 5 × 10^5^ BMMs per well of a 12-well plate using the DNA Mini Prep Plus Kit (#D4069, Zymo Research, Irvine, USA), following the manufacturer's instructions. Mitochondrial DNA (mtDNA) copy number was determined by quantitative real-time PCR (qRT-PCR), measuring the expression levels of mitochondrial genes *mt16S* and *mtND1*, with *Hk2* as the nuclear reference gene. Primer sequences are provided in Supplemental Table 1. The mtDNA copy number was calculated as the ratio of mtDNA content (mean expression of *mt16S* and *mtND1*) to genomic DNA (gDNA), represented by *Hk2* expression. This method was adapted from the protocol described by Quiros et al. (2017).

### Measurement of Mitochondrial Function

MitoTracker™ Deep Red and MitoTracker™ Green (#M22426 and #M7514, respectively; Invitrogen, Carlsbad, USA) were prepared according to the manufacturer's instructions. BMMs were seeded in 6-well plates at a density of 1 × 10^6^ cells per well. The following day, cells were incubated with 100 nM MitoTracker™ Green and MitoTracker™ Deep Red for 30 minutes at 37°C. Untreated controls were included. Cells were detached using a cell scraper (TPP, Trasadingen, Switzerland), washed, and resuspended in phosphate-buffered saline (PBS) (2.7 mM KCl, 1.8 mM KH_2_PO_4_, 137 mM NaCl, 10 mM Na_2_HPO_4_, pH 7.4). Flow cytometry was performed on an LSR-Fortessa™ (BD Biosciences) using FACS Diva 8.0.1 software (BD Biosciences, Allschwil, Switzerland). A minimum of 1 x 10^4^ events were recorded per sample. Data analysis and pseudocolor plot generation were performed using FlowJo 10.10.0.

### Measurement of Mitochondrial Superoxide

To assess mitochondrial reactive oxygen species (ROS) production, cells were incubated with 5 µM MitoSOX™ Red mitochondrial superoxide indicator (#M36008, Invitrogen, Carlsbad, USA) in PBS containing 2% FBS. Antimycin A (AA, 10 µM) was used as a positive control and added simultaneously. BMMs were seeded at a density of 5 × 10^4^ cells per well in 96-well plates and incubated overnight. Following treatment, real-time imaging was performed using the Incucyte^®^ S3 Live-Cell Analysis System (Essen BioScience, Ann Arbor, USA) with brightfield and red fluorescence channels (10× objective, 400 ms acquisition time). The total integrated intensity was measured after 15 minutes of treatment and normalized to the cell confluence (%) at the start of the experiment to account for variations in cell density.

### Western Blot

For Western blot analysis, BMMs were seeded at 1 × 10^6^ cells per well in a 6-well plate and incubated overnight. Western blots for IMP2/p62 were performed as previously described using antibodies specific for IMP2/p62 25,26. Western blot for phosphor-p44/42 MAPK (Thr202/Tyr204, 20G11, #4376S, Cell Signaling, Danvers, USA) and p44/42 MAPK (Erk1/2, L34F12, #4696S, Cell Signaling, Danvers, USA), phospho-NF-κB p65 (Ser536, 93H1, #3033S, Cell Signaling, Danvers, USA), and NF-κB p65 (L8F6, #6956S, Cell Signaling, Danvers, USA) were performed as previously described 27. An anti-α-tubulin monoclonal antibody (DM1A, #T9026, Sigma-Aldrich, St. Louis, USA) was used to detect α-tubulin, which served as a loading control 25-27.

Immobilon FL-PVDF membranes (#IPFL00010, Millipore, Billerica, USA) were blocked for 1.5 hours in Rockland blocking buffer for near-infrared Western blotting (#MB-070, Rockland Immunochemicals, Limerick, USA). Following incubation overnight at 4 °C with primary antibody dilutions (1:1000 in Rockland blocking buffer) and incubation for 1.5 hours at room temperature with IRDye 680- or IRDye 800- (#926-68071, #926-32210, LI-COR Biosciences, Lincoln, USA) conjugated secondary antibodies (1:10,000 in Rockland blocking buffer). After final washes, signals were detected and quantified using an Odyssey imager and Image Studio software (LI-COR Biosciences, Lincoln, USA).

### qRT-PCR

BMMs were seeded at 5 × 10^5^ cells per well for RNA isolation in 12-well plates. Total RNA was isolated using the High Pure RNA Isolation Kit (#11828665001, Roche, Basel, Switzerland) or the Total RNA Quick-RNA Miniprep Kit (#R1055, Zymo Research, Irvine, USA) according to the manufacturer's protocols. The concentration and purity of the isolated RNA were assessed using a NanoDrop™ spectrophotometer (Thermo Fisher Scientific, Waltham, USA). Equal amounts of RNA were reverse transcribed into complementary DNA (cDNA) using the High-Capacity cDNA Reverse Transcription Kit (#4368813, Thermo Fisher Scientific, Waltham, USA) with the addition of an RNase inhibitor (Invitrogen; #10777-019), as per the manufacturer's instructions. Quantitative real-time PCR (qRT-PCR) was performed using 5× HotFirePol EvaGreen qPCR Plus Mix (no ROX; #082500020, Solis BioDyne, Tartu, Estonia), as described previously 28,29, with primers listed in Supplemental Table 1. Reactions were conducted in a CFX96 Touch™ Real-Time PCR Detection System (Bio-Rad, Hercules, USA).

Each run included no-template controls (NTCs). Specificity was verified for every run by melting-curve analysis. During assay establishment, selected assays were further verified by agarose gel electrophoresis (to confirm the expected amplicon size) or by Sanger sequencing of the corresponding standard plasmids (Eurofins Genomics, Ebersberg, Germany). For absolute quantification, plasmid standards for the gene of interest and housekeeping gene were used to generate standard curves; copy numbers were interpolated and expressed as the ratio (target/housekeeper). Alternatively, relative quantification (ΔΔCq) was used, and assay efficiency was validated using a cDNA dilution series. Gene expression data were normalized to the housekeeping genes *RNA18S* or *Ppia* (as indicated).

### Enzyme-Linked Immunosorbent Assay (ELISA)

BMMs were seeded in 96-well plates at a density of 5 × 10^4^ cells per well and incubated overnight. The following day, cells were treated with 100 ng/ml LPS for 4 hours. After incubation, supernatants were collected for analysis. Following the manufacturer's protocol, tumor necrosis factor (TNF)-α secretion was quantified using an ELISA kit (#430904, BioLegend, San Diego, USA).

### Griess Assay

BMMs were seeded in 96-well plates at a density of 5 × 10^4^ cells per well the day before treatment. The following day, cells were treated with 100 ng/ml LPS and 25 ng/ml IFN-γ. After 24 hours of incubation, nitrite, a stable metabolite of nitric oxide (NO), was quantified.

For the assay, 90 μl of 1% sulfanilamide in 5% phosphoric acid (H₃PO₄) and 90 μl of 0.1% N-(1-naphthyl) ethylenediamine dihydrochloride in water were added to 100 μl of cell culture supernatant. The mixture was incubated at room temperature for 10 minutes, and the absorbance was measured at 560 nm using a GloMax^®^ Discover Microplate Reader (Promega, Madison, USA). As described previously, a standard curve was generated using sodium nitrite (NaNO₂) on the same plate for quantification 30.

### Lipidomic Analysis of BMMs

BMMs were seeded in a 25 cm² cell culture flask at a density of 2 × 10^6^ cells in 5 ml of medium one day before treatment. The following day, M0 macrophages were maintained in a standard medium, and TAM-like macrophages were generated by culturing macrophages for 24 hours in the LLC1-conditioned supernatant. Cell pellets were then collected and stored for further analysis.

For quantitative lipidomics, internal standards were added prior to lipid extraction. An amount of cellular material containing 100 μg protein was subjected to lipid extraction according to the protocol by Bligh and Dyer 31.

The analysis of lipids was performed by direct flow injection analysis (FIA) using a triple quadrupole mass spectrometer Xevo TQ-S micro (Waters, Milford, USA; FIA-MS/MS) and a high-resolution hybrid quadrupole-Orbitrap mass spectrometer QExactive (Thermo Fisher Scientific, Bremen, Germany; FIA-FTMS). FIA-MS/MS was performed in positive ion mode using the analytical setup and strategy described previously 32. A fragment ion of *m/z* 184 was used for lysophosphatidylcholines (LPC) 33. The following neutral losses were applied: Phosphatidylethanolamine (PE) and lysophosphatidylethanolamine (LPE) 141, phosphatidylserine (PS) 185, phosphatidylglycerol (PG) 189, and phosphatidylinositol (PI) 277 34. Sphingosine-based ceramides (Cer) and hexosylceramides (Hex-Cer) were analyzed using a fragment ion of *m/z* 264 35. PE-based plasmalogens (PE-P) were analyzed according to the principles described by Zemski-Berry 36. Cardiolipin (CL) was monitored by diglycerol fragment ions 37. Glycerophospholipid species annotation assumed of even-numbered carbon chains only.

A detailed description of the FIA-FTMS method was published recently 38. Triglycerides (TG), diglycerides (DG), and cholesterol esters (CE) were recorded in positive ion mode *m/z* 500-1000 as [M+NH_4_]^+^ at a target resolution of 140,000 (at 200 *m/z*). CE species were corrected for their species-specific response 39. Phosphatidylcholines (PC), PC ether (PC O), and sphingomyelins (SM) were analyzed in negative ion mode* m/z* 520-960 as [M+HCOO]^-^ at the same resolution setting. Analysis of free cholesterol (FC) was performed by multiplexed acquisition (MSX) of the [M+NH_4_]^+^ of FC and the deuterated internal standard (FC[D7]) 39. Free fatty acids (FA) were analyzed in negative ion mode* m/z* 150-450 as [M-H]^-^ dissolved in methanol/chloroform = 5/1 (v/v) containing 0.005% dimethylamine.

### Migration Measurements

Migration was analyzed using the IncuCyte^®^ S3 system. BMMs were seeded at a density of 1 x 10^5^ cells per well in an ImageLock 96-well plate. Scratches were created using the WoundMaker^®^ tool (IncuCyte Migration Kit). Following scratch creation, cells were washed twice with medium to remove debris. Cell migration was monitored for 4 hours under standard incubation conditions. The extent of scratch area coverage (%) was assessed by analyzing the cell-covered area using IncuCyte migration software. Consistent with the non-proliferative nature of BMMs, control experiments confirmed that proliferation contributed negligibly (< 0.5 % over 4 h) to scratch closure Supplemental Figure 1. Quantification was used to determine the migration rate and the extent of scratch area coverage over time.

### Dorsal Skinfold Chamber and LPS-Induced Striated Skin Muscle Inflammation

WT and IGF2BP2 ΜΦ-KO mice (mean age: 5 months; average body weight: 25 g) were anesthetized *via* intraperitoneal injection of ketamine (100 mg/kg) and xylazine (12 mg/kg), followed by dorsal skinfold chamber implantation.

Briefly, two symmetrical titanium frames were positioned on the extended dorsal skinfold of anesthetized mice to create a double-layered skinfold. One layer, consisting of the skin, subcutis, and retractor muscle, was removed entirely within a circular area of 15 mm in diameter. This exposed area was then covered with a removable cover slip and secured with a snap ring, allowing direct microscopic access to the microcirculation within the chamber. Following the procedure, all animals were allowed to recover for 48 hours.

After the recovery period, the chamber tissue of anesthetized WT and IGF2BP2 ΜΦ-KO mice was topically treated for 0.5 hours with LPS (10 µg/ml; L2887, Sigma-Aldrich, St. Louis, USA), prepared as a 2 mg/ml stock solution in Aqua dest. (#B230673, Fresenius, Bad Homburg v.d.H., Germany) and diluted in 0.9% NaCl (X92900.1, Fresenius, Bad Homburg v.d.H., Germany), to induce local inflammation. Briefly, anesthetized mice were immobilized on a plexiglas stage, and the dorsal skinfold chamber was affixed to the microscopic stage. To enhance contrast, retrobulbar intravenous injections of 0.05 ml 5% fluorescein isothiocyanate (FITC)-labeled dextran (150,000; dissolved in 0.9% NaCl) were administered to stain blood plasma, along with 0.05 ml 0.1% rhodamine 6G to stain leukocytes.

Intravital fluorescence microscopy was performed to assess leukocyte-endothelial interactions, blood vessel diameters, and macromolecular leakage under baseline conditions (19 hours before LPS exposure) and at 0.5, 3, and 24 hours post-LPS treatment, as previously described 40. Leukocyte rolling and adhesion were quantified using the established methods described 40,41. Vessel diameters were measured in micrometers (µm).

### LLC1-Tumor Model

A murine cancer model was established by subcutaneous injection of luciferase-expressing Lewis lung carcinoma (LLC1) cells (CRL-1642-LUC2™, ATCC, Manassas, USA) into 8- to 10-week-old female WT and IGF2BP2 ΜΦ-KO mice. The LL2-Luc cell line was authenticated for viability, growth characteristics, mycoplasma contamination, species identity, and sterility. Cells were cultured under standard conditions according to ATCC guidelines. Before injection, subconfluent LLC1 cells were maintained for 2-3 weeks, harvested, filtered through a 40-μm cell strainer (BD Biosciences, Allschwil, Switzerland), washed, and resuspended in PBS. Tumor induction was performed by subcutaneous injection of 5 × 10^5^ cells into the right flank of each mouse. Tumor growth was monitored by measuring tumor dimensions with digital calipers, and volumes were estimated using the formula: (height × width²)/2.

Bioluminescence imaging (BLI) was conducted on day 14 post-injection to assess tumor burden. Mice were administered 3 mg of D-luciferin potassium salt (#122799, Revvity, Waltham, USA) *via* subcutaneous injection. Luciferin was prepared according to the manufacturer's instructions (100 mg luciferin in 3.33 ml 1x DPBS (#15326239, Thermo Fisher Scientific, Waltham, USA), and bioluminescent signals were captured using the IVIS Spectrum in vivo imaging system (PerkinElmer, Waltham, USA). Image acquisition and quantification were performed using Living Image 4.5 software (PerkinElmer, Waltham, USA).

### Flow Cytometry

Tumor cells were isolated from murine tumor tissue on day 14. Tumors were enzymatically dissociated using the Murine Tumor Dissociation Kit (#130-096-730, Miltenyi Biotec, Bergisch Gladbach, Germany) and the gentleMACS Octo Dissociator (Miltenyi Biotec, Bergisch Gladbach, Germany) according to the manufacturer's instructions. Following dissociation, cells were washed with PBS and stained with Zombie Yellow viability dye (#423101, BioLegend, San Diego, USA) for 20 minutes at room temperature in the dark. A blocking step was performed using BD Fc Block™ (#553142, BD Biosciences, Allschwil, Switzerland) for 10 minutes at room temperature. Cells were incubated with fluorophore-conjugated antibodies for extracellular staining in FACS Wash buffer (PBS containing 2.5% FBS and 0.05% sodium azide). The following antibodies were used: APC anti-mouse/human CD11b (Clone M1/70, #101212, 2 µg/ml, BioLegend, San Diego, USA), PE anti-mouse F4/80 (Clone BM8, #123110, 2.5 µg/ml, BioLegend, San Diego, USA), APC anti-mouse CD206 (MMR) (Clone C068C2, #141707, 2.5 µg/ml, BioLegend, San Diego, USA), Brilliant Violet 421™ anti-mouse CD86 (Clone GL-1, 2 µg/ml, #105031, BioLegend, San Diego, USA), APC anti-mouse CD4 (Clone RM4-5, #100515, 2 µg/ml, BioLegend, San Diego, USA), Brilliant Violet 421™ anti-mouse CD8a (Clone 53-6.7, #100737, 5 µg/ml, BioLegend, San Diego, USA), and PE anti-mouse NK-1.1 (Clone PK136, #108707, 2 µg/ml, BioLegend, San Diego, USA). After staining, cells were washed and resuspended in formaldehyde (FA) (1%) in PBS.

For intracellular CD68 staining, cells were fixed in FA (1%) for 30 minutes at 4 °C, followed by permeabilization with saponin buffer (FACS Wash with 0.2% [w/v] saponin) for 10 minutes. Subsequently, cells were blocked in PBS supplemented with 20% FBS and 0.02% saponin for 30 minutes, then incubated with Alexa Fluor^®^ 594 anti-mouse CD68 (Clone FA-11, #137020, 5 µg/ml, BioLegend, San Diego, USA) for an additional 30 minutes.

Flow cytometric analysis was performed using a BD LSRFortessa™ (BD Biosciences, Allschwil, Switzerland). Data acquisition and analysis were conducted using BD FACSDiva™ software (BD Biosciences, Allschwil, Switzerland), and median fluorescence intensities of singlet cells were used to quantify surface marker expression.

### Immunohistochemistry

Tumor tissues were fixed in FA (1%) at 4 °C for 24 hours. Following fixation, the samples were dehydrated, embedded in paraffin, and sectioned into 3-μm-thick slices. The tissue sections were treated with citrate buffer (pH 6.0) for antigen retrieval at 95 °C for 30 minutes. To block non-specific binding, sections were incubated with 3% goat serum (Jackson ImmunoResearch, Ely, United Kingdom) for 1 hour at room temperature. CD31 expression was detected by incubating the sections with a primary antibody against CD31 (1:300, Clone SZ31, #DIA310, Dianova, Hamburg, Germany) overnight at 4°C. The primary antibody was visualized using a Cy3-conjugated goat anti-rat secondary antibody (1:1000, #712-165-153, Jackson ImmunoResearch, Ely, United Kingdom), applied for 1 hour at room temperature, as previously described 42. Fluorescence imaging was performed using a BX60F fluorescence microscope (Olympus, Tokyo, Japan) equipped with an Olympus DP73 camera (Olympus, Tokyo, Japan), and CD31-positive cells were quantified using FIJI software (NIH).

### m^6^A Motif Enrichment Analysis

To investigate whether DEGs in IGF2BP2 KO macrophages are enriched for m^6^A consensus motifs, a motif enrichment analysis was conducted using the MEME Suite tool SEA 43. Bulk RNA-Seq data from WT and IGF2BP2 KO BMMs were analyzed under untreated and IL-4-stimulated conditions. DEGs were defined using DESeq2 (padj < 0.05, |log2FC| ≥ 2). The 3' untranslated regions (3'UTRs) of significantly up- and downregulated DEGs were extracted from the Gencode M10 annotation 44,45, with non-differentially expressed genes serving as controls.

Enrichment of the canonical m^6^A motif (DRACH 46) was assessed using the Simple Enrichment Analysis (SEA^43^) tool within the MEME Suite, and motif occurrences were identified using FIMO (Find Individual Motif Occurrences) 47. Only motifs with a p-value < 1e-4 (uncorrected) were considered for further analysis.

### Statistics

Data are presented as means ± SEM (bar graphs). The normality of the data distribution was assessed using the Shapiro-Wilk test. A Student's t-test was applied for normally distributed data for comparisons between two groups, while the Mann-Whitney U test was used for non-normally distributed data. Statistical significance across multiple groups was determined by one-way ANOVA (for single time point comparisons) and two-way ANOVA (for multiple time points), followed by Bonferroni post-hoc tests for normally distributed data. Outliers were identified using Grubbs' test.

Unless stated otherwise, all statistical analyses and visualizations were performed using OriginPro 2020b (OriginLab, Northampton, MA, USA).

## Results

### Differentially Expressed Genes in WT and IGF2BP2 KO Macrophages

**Figure 1:**
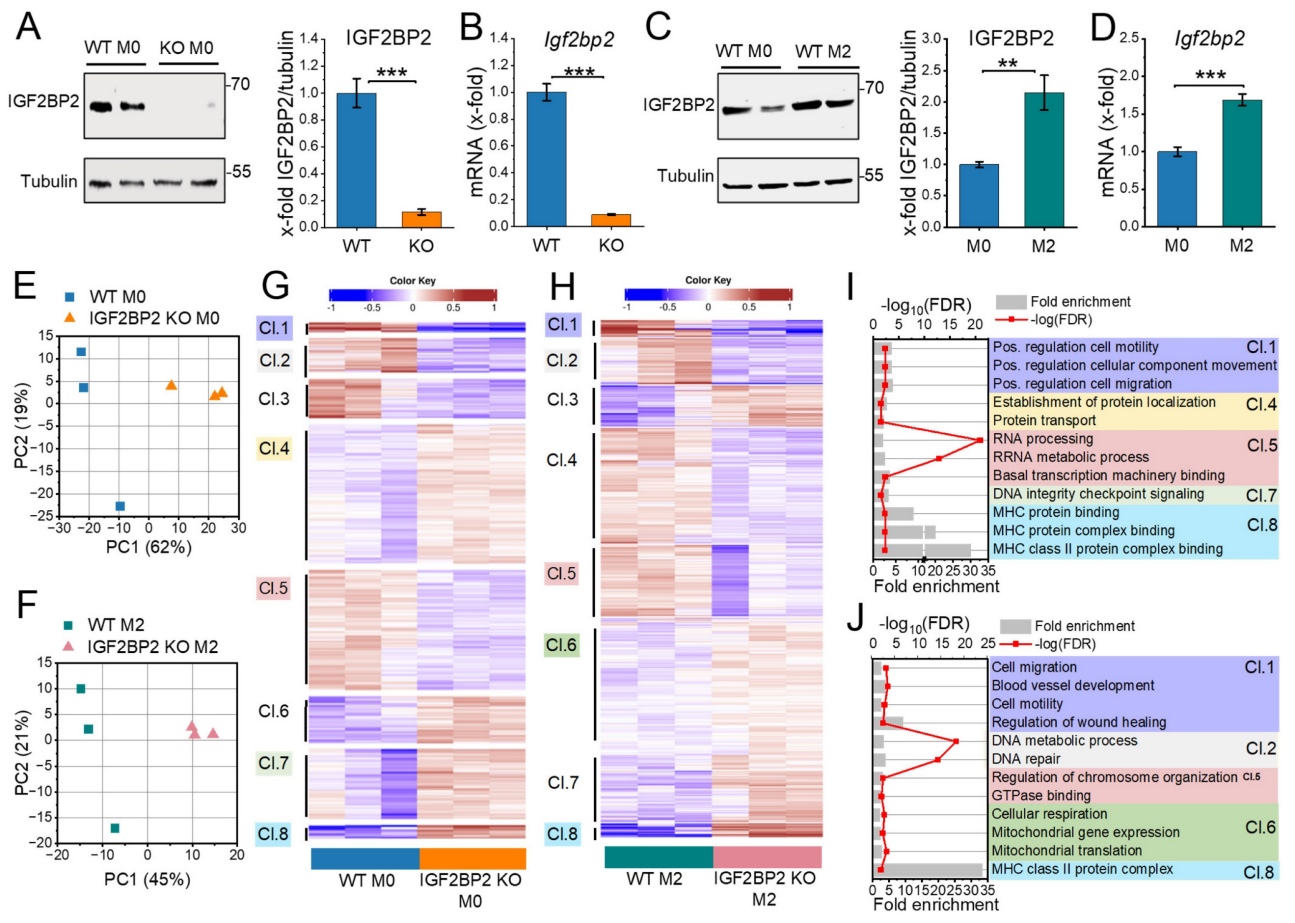
** Differentially expressed genes in WT and IGF2BP2 KO macrophages.** BMMs from WT and IGF2BP2 ΜΦ-KO mice were cultured in standard medium (M0) or treated with IL-4 (8 h, 20 ng/ml) to induce M2 polarization. **(A-D)** IGF2BP2 expression was assessed at the **(A, C)** protein level (n = 10 mice per group; n = 3 mice per group, duplicates), normalized to tubulin, and **(B, D)** mRNA level (n = 3 mice per group; n = 4 mice per group, duplicates), normalized to *RNA18S* (B) or *Ppia* (D). Data are expressed as fold change relative to untreated WT M0. **(E, F)** PCA of transcripts per million (TPM) values for all annotated protein-coding genes (>0.5 TPM; n = 3 mice per group). **(G, H)** K-means clustering of DEGs identified by DESeq2 analysis (input data = TPM) in **(E, G)** untreated and **(F, H)** IL-4-treated BMMs. **(I, J)** Selected Gene Ontology (GO) terms for biological processes, molecular functions, and cellular components enriched in the clusters (Cl.) identified in **(G, H)**.** I** is based on clustering from **G,** while **J** is based on clustering from **H**. Fold enrichment values are shown, and enrichment significance is represented by false discovery rate (FDR) values (fold change > 2, FDR < 0.05). **(A-D)** Data are presented as mean ± SEM. Statistical analysis was performed using Student's *t*-test (*p < 0.05; **p < 0.01; ***p < 0.001).

Our previous study 20 demonstrated that IGF2BP2 expression is upregulated in human M2 macrophages (ΜΦ) following interleukin-4 (IL-4) treatment. To characterize the impact of IGF2BP2 expression in an anti-inflammatory context, we generated mice with a myeloid-specific deletion of IGF2BP2. Bone marrow-derived macrophages (BMMs) from IGF2BP2 MΦ-knockout (KO) mice showed a significant reduction in *Igf2bp2* expression to near-undetectable levels at both the protein and mRNA levels compared to wild-type (WT) BMMs (Fig. 1A, B). Consistently, IL-4 treatment of WT BMMs led to increased *Igf2bp2* expression at both the mRNA and protein levels (Fig. 1C, D).

We performed RNA-Seq with WT and IGF2BP2 KO BMMs under untreated conditions and after IL-4 treatment. Using the DESeq2 approach, we identified 2,739 differentially expressed genes (DEGs, p < 0.05) in untreated cells and 2,136 in IL-4-treated cells Supplementary file S1). Principal component analysis (PCA) revealed clear genotypic grouping patterns closely linked to differences in gene expression between WT and IGF2BP2 KO BMMs (Fig. 1E, F).

We then performed hierarchical k-means clustering (Fig. 1G, H) to analyze the biological processes associated with the loss of IGF2BP2 in untreated and IL-4-treated cells. Figures 1I and 1J show representative gene ontology (GO) terms for biological processes for selected clusters (fold change > 2, FDR < 0.05), providing insights into the functional categories affected by IGF2BP2 expression (see Supplementary file S2 for the complete list). Notably, our analysis revealed significant metabolic alterations in IGF2BP2 KO macrophages. Specifically, genes associated with metabolic processes and mitochondrial function, including cellular respiration and mitochondrial translation, were upregulated in IL-4-treated IGF2BP2 KO macrophages (Cluster 6). Additionally, KEGG pathway analysis identified an increase in glycolysis and gluconeogenesis (Supplementary Fig. 2A, B). Differential pathway analysis using Ingenuity Pathway Analysis (IPA) further confirmed the upregulation of metabolic pathways in KO macrophages, including glycolysis and gluconeogenesis (Supplementary Fig. 2C, D).

### Metabolic Reprogramming in IGF2BP2 KO Macrophages: Increased Glycolysis and Impaired Mitochondrial Function

**Figure 2:**
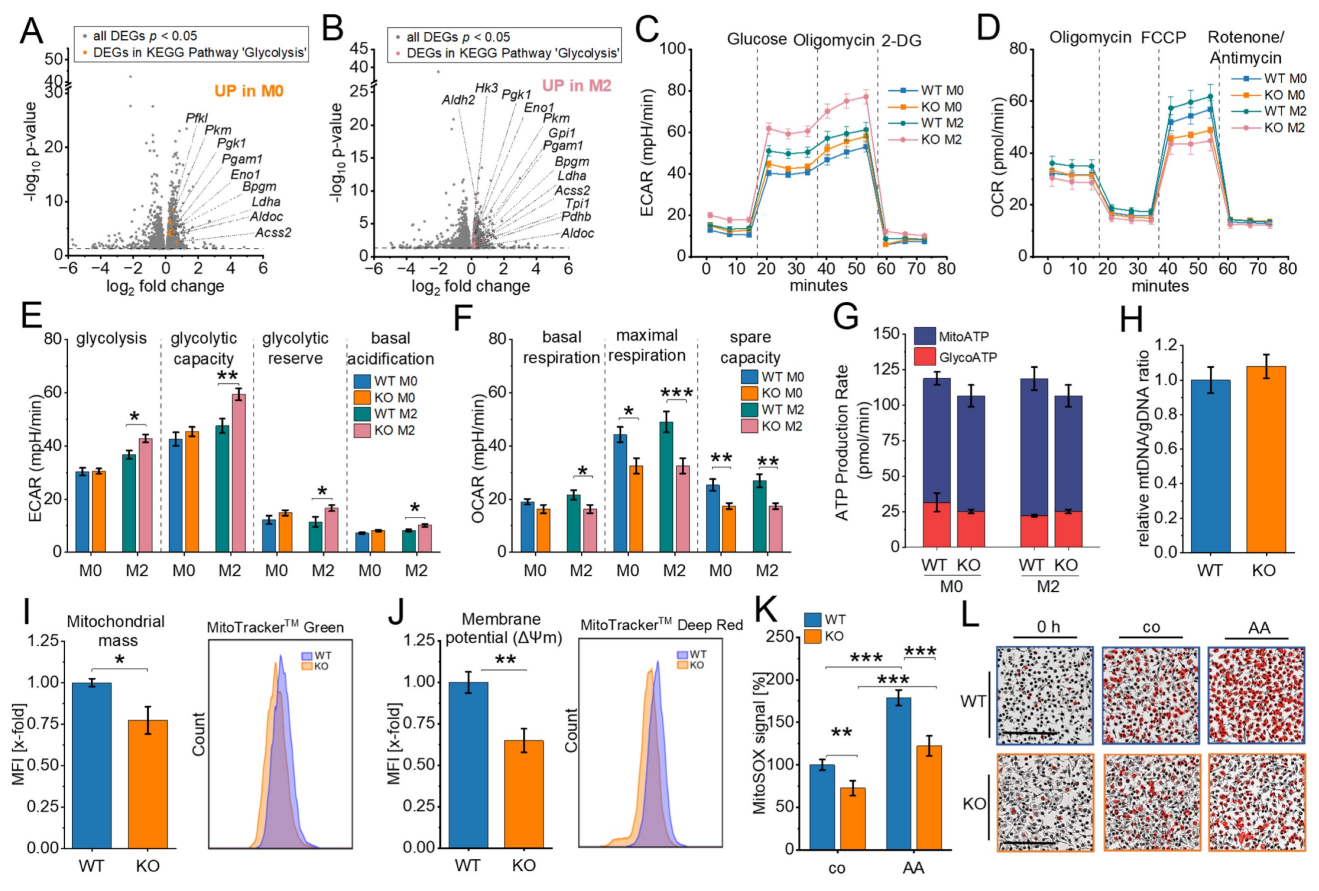
** Metabolic reprogramming in IGF2BP2 KO macrophages. (A, B)** Volcano plots of DEGs (shown in gray), with significantly upregulated glycolytic pathway genes highlighted in M0 (orange, A) and M2 (purple, B) macrophages (n = 3 mice per group). **(C, E)** Extracellular acidification rate (ECAR) during the glycolysis stress test and **(D, F)** Oxygen consumption rate (OCR) during the mitochondrial stress test in untreated (M0) and IL-4-treated BMMs, measured using the Seahorse XF Pro Analyzer (n = 4 mice per group, triplicates). **(H)** Quantitative RT-PCR detection of the relative mtDNA/gDNA ratio in BMMs (n = 8 mice per group, duplicates). **(I, J)** Flow cytometric measurements of mitochondrial mass **(I)** and mitochondrial membrane potential **(J)** relative to WT BMMs (n = 5-6 mice per group, duplicates), with pseudocolor plots of representative samples. **(K, L)** The MitoSOX™ signal was measured using live-cell imaging with the Incucyte^®^ S3 system 15 minutes after adding MitoSOX or Antimycin A (AA). Fluorescence intensities relative to WT (set to 100%) are shown. **(L)** Representative Images. The fluorescence signal is shown in red. Scale bar = 200 µm (n = 4 mice per group, quadruplicates). *p* values were determined using one-way ANOVA followed by Bonferroni post-hoc tests (E-G, K) or Student's *t*-test (I, J) (* *p* < 0.05; **p < 0.01; ***p < 0.001).

Genes within the glycolytic pathway were significantly upregulated in both M0 and M2 macrophages in IGF2BP2 KO cells (Fig. 2A, 2B; Supplementary Fig. 2A), with nine genes upregulated in M0 macrophages and thirteen genes upregulated in M2 macrophages. To assess the functional implications of these transcriptomic changes, we evaluated glycolytic activity by measuring the extracellular acidification rate (ECAR) using the Seahorse Glycolytic Stress Test. The results showed a significant increase in glycolysis, glycolytic capacity, and glycolytic reserve in M2 KO macrophages (Fig. 2C, E). These results suggest an increased reliance on glycolysis for energy production in IGF2BP2 KO macrophages.

Cells can dynamically switch between glycolysis, taking place in the cytosol, and oxidative phosphorylation (OXPHOS), localized within mitochondria, to meet energy demands (48. Given that metabolic homeostasis appears to be disrupted in KO cells, we further examined mitochondrial function by assessing their oxygen consumption rate (OCR). In line with the observed increase in glycolytic activity, IGF2BP2 KO macrophages exhibited a significant reduction in maximal respiration and reserve respiratory TAM- capacity in both untreated and M2 KO conditions (Fig. 2D, F). No significant differences were observed in basal respiration or ATP production rates in KO macrophages (Fig. 2F, G), indicating a specific impairment of mitochondrial respiratory capacity rather than overall energy production. Despite these functional changes, the mitochondrial DNA (mtDNA) content remained unchanged, and only a slight reduction in mitochondrial mass was observed (Fig. 2H, I). The mitochondrial membrane potential (Δψm), driven by the proton gradient generated during OXPHOS, was also decreased in IGF2BP2 KO macrophages, consistent with the observed reduction in OXPHOS activity (Fig. 2J). Moreover, mitochondrial superoxide generation, i.e., reactive oxygen species (ROS) produced during electron flow through the electron transport chain (ETC), was significantly reduced (Fig. 2K, L). This decrease reflects the lower electron flow associated with reduced OXPHOS through ETC. Transcriptomic analysis further revealed the upregulation of genes involved in mitochondrial translation and mitochondrial organization in IGF2BP2 KO macrophages. Among these, genes encoding components of the ETC were significantly increased Supplementary Fig. 2B, C), potentially representing a compensatory mechanism to counterbalance the reduced mitochondrial function.

### IGF2BP2 Deficiency Alters TAM-like Polarization and Metabolic Reprogramming

**Figure 3:**
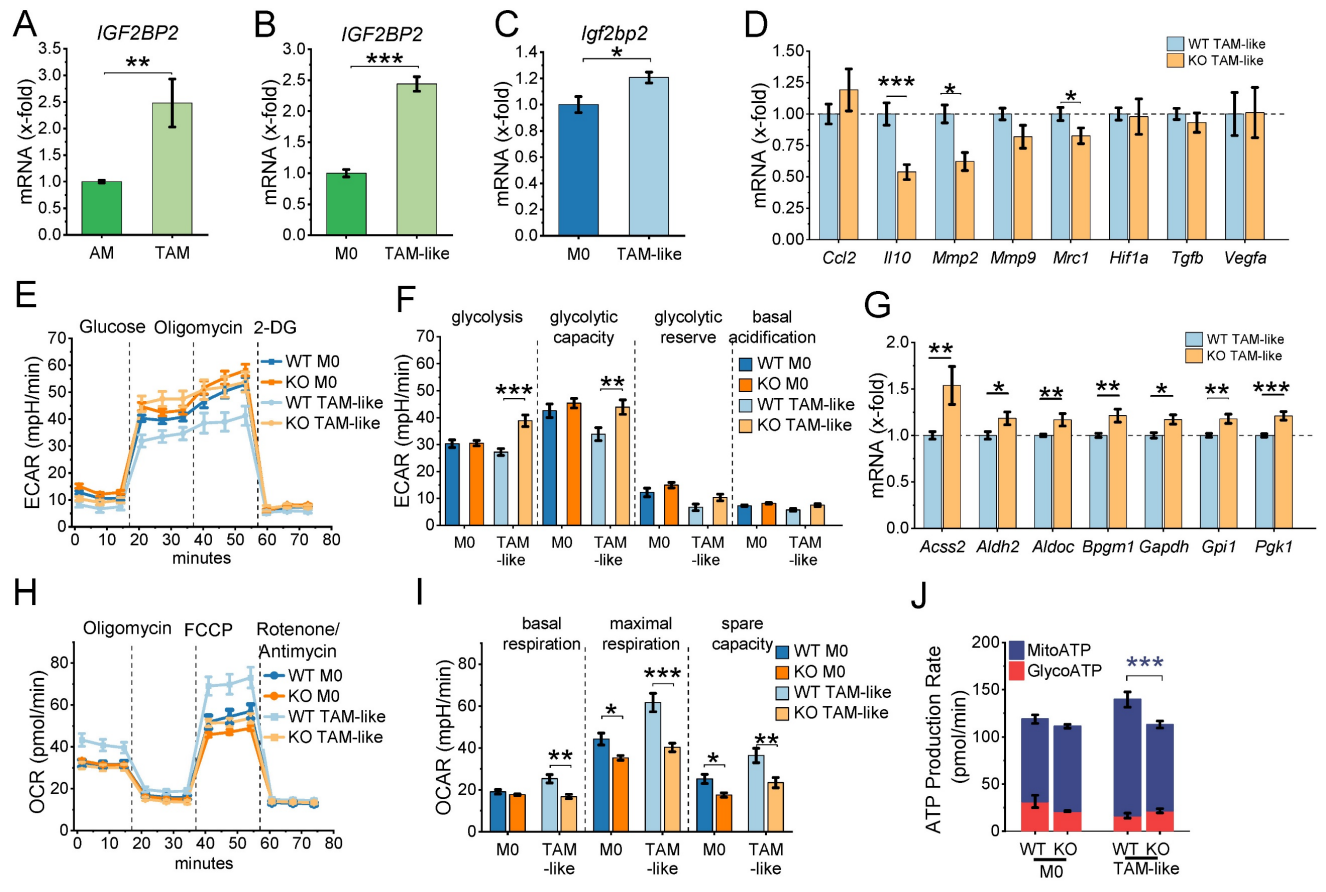
** IGF2BP2 regulates TAM-like polarization and metabolic reprogramming. (A)** Gene expression in macrophages from lung adenocarcinoma (TAMs) and adjacent non-tumor tissue (AMs) using publicly available RNA-Seq data from dataset GSE162669 (n = 3 donors per group, triplicates). **(B)** Gene expression of HMDMs polarized with TCM from A549 cells for 24 h, using publicly available data from GSE162698 (n = 3 individual donors). **(C-J)** BMMs were cultured in standard medium (M0) or TCM from LLC1 cells (TAM-like) for 8 hours. mRNA expression was measured by qPCR and normalized to *Ppia*. **(C)** *Igf2bp2* expression is presented as fold change relative to M0 (n = 4 mice per group, duplicates). **(D)** Expression of TAM-like macrophage markers is shown as a fold change of WT TAM-like (n = 6 mice per group, duplicates). **(E, F)** ECAR during the Glycolysis Stress Test and **(H-J)** OCR during the mitochondrial stress test were measured in M0 and TAM-like macrophages using the Seahorse XF Pro Analyzer (n = 4 mice per group, triplicates). **(G)** mRNA expression of glycolytic markers, expressed as x-fold change relative to TAM-like macrophages (n = 4 mice per group, duplicates). Data are presented as mean ± SEM. Statistical significance was determined by Student's *t*-test (A-D, G) or ANOVA followed by Bonferroni post-hoc test (F, I, J) (*p < 0.05; **p < 0.01; ***p < 0.001).

Analysis of publicly available transcriptomic data (GSE162669, GSE162698; (22) indicates that IGF2BP2 expression is elevated in TAMs isolated from lung adenocarcinoma tissue compared to alveolar macrophages (AMs) from adjacent normal lung tissue (Fig. 3A). A similar upregulation was observed in TAM-like macrophages, where primary human monocyte-derived macrophages (HMDMs) were polarized towards a TAM-like phenotype using tumor-conditioned medium (TCM) from the lung adenocarcinoma cell line A549 (Fig. 3B).

To further explore the role of IGF2BP2 in murine TAM polarization, BMMs were stimulated with TCM from Lewis lung carcinoma (LLC1) cells to induce a TAM-like phenotype. qPCR analysis revealed an upregulation of *Igf2bp2* mRNA after TAM-like polarization (Fig. 3C). A panel of TAM-associated genes was analyzed by qPCR, revealing increased expression of several genes linked to TAMs (*Ccl2, Hif1a, Mmp9, Mrc1, Tgfb, Vegfa*) upon polarization Supplement Fig. 4. Notably, IGF2BP2 KO BMMs exhibited a significant reduction in selected TAM-associated genes, including *Mrc1*, *Mmp2*, and *Il10* (Fig. 3D). To evaluate how closely the TCM-based TAM-like macrophage model reflects the gene expression pattern of tumor-associated macrophages *in vivo*, we compared selected marker genes with RNA-Seq data from human TAMs and alveolar macrophages (GSE162669). As shown in Supplemental Figure 5, the murine TAM-like macrophages partially recapitulated the TAM expression profile, including increased expression of *Vegfa*, *Ccl2*, and *Mrc1*, whereas other genes, such as *Il10* and *Mmp2*, showed divergent regulation. These findings indicate that TCM treatment induces a TAM-associated, but not fully tumor-matched, macrophage phenotype.

Given the metabolic shift observed in IGF2BP2 KO M2 macrophages, we assessed the glycolytic activity in KO TAM-like macrophages using the Glycolytic Stress Test. ECAR measurements indicated a significant increase in glycolysis and glycolytic capacity in KO TAM-like macrophages compared to WT TAM-like macrophages (Fig. 3E, F). Consistent with elevated glycolytic activity, qPCR analysis demonstrated slight upregulation of glycolytic markers, including *Accs2*, *Aldh2*, *Aldoc*, *Bpgm1*, *Gapdh*, *Gpi1*, and *Pgk1*, in KO TAM-like macrophages (Fig. 3G). Mitochondrial respiration was evaluated using the Seahorse XF Cell Mito Stress Test. KO TAM-like macrophages exhibited a significant reduction in maximal respiration and reserve respiratory capacity (Fig. 3H-J). Furthermore, ATP production analysis revealed a decrease in mitochondrial-driven ATP (MitoATP) levels in KO TAM-like macrophages (Fig. 3J).

### Lipidomic Profiling Reveals Altered Membrane Lipid Composition in IGF2BP2 KO Macrophages

Lipid metabolism plays a crucial role in shaping the phenotype and function of TAMs 49. To explore the lipidomic changes associated with TAM polarization and assess the impact of IGF2BP2 on macrophage lipid metabolism, we conducted a comprehensive lipidomic analysis of WT and IGF2BP2 KO macrophages in both their native M0 and TAM-like states Supplementary file S3). TAM-like macrophages were generated by culturing BMMs in TCM from LLC1 cells for 24 hours.

**Figure 4:**
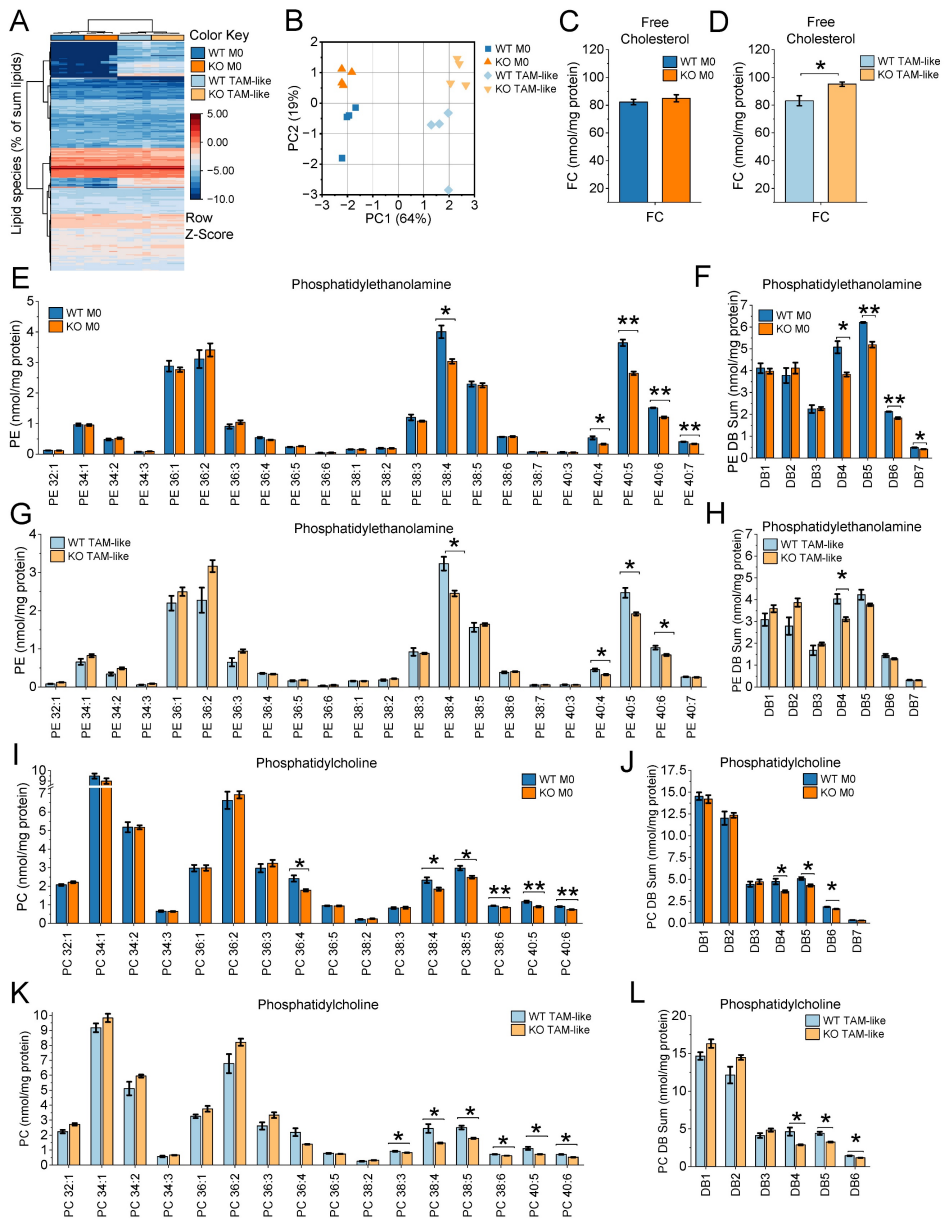
** IGF2BP2 deficiency alters membrane lipid composition in macrophages (M0 and TAM-like).** BMMs were cultured either in standard medium (M0) or in TCM from LLC1 cells for 8 hours to induce TAM-like polarization. Lipid concentrations in WT and IGF2BP2 KO M0 and TAM-like macrophages were analyzed by mass spectrometry. **(A)** Hierarchical clustering of log₂-transformed lipid data expressed as a percentage of total lipids, using Ward's linkage and Euclidean distances. Samples are color-coded: WT M0 (dark blue), KO M0 (orange), WT TAM-like (light blue), and KO TAM-like (yellow). **(B)** PCA of log₂-transformed lipid concentrations (nmol/mg protein) across all lipid species. **(C, D)** Quantification of free cholesterol (FC) in WT and IGF2BP2 KO macrophages under M0 and TAM-like conditions. **(E-H)** Concentrations of phosphatidylethanolamine (PE) species (E, G) and PE species grouped by double bond (DB) number (F, H). **(I-L)** Concentrations of phosphatidylcholine (PC) species (I, K) and PC species grouped by DB number (J, L). Lipid concentrations are expressed as nmol/mg protein. Statistical significance was determined by Student's t-test (C, D) or ANOVA with Bonferroni's post hoc test (E-J). (*p < 0.05; **p < 0.01; ***p < 0.001).

Lipidomic profiling revealed consistent, though moderate, alterations between WT and IGF2BP2 KO macrophages. In the heatmap and PCA (Fig. 4A, B, Supplementary Fig. 6, WT and KO samples segregated into distinct groups under both M0 and TCM-treated conditions, indicating genotype-dependent differences in lipid composition. TAM-like polarization induced changes in glycerolipid content, especially a moderate increase in triglycerides consistent with enhanced lipid storage 50, the overall distribution of major lipid classes remained relatively comparable between WT and IGF2BP2 KO macrophages Supplementary Fig. 6A, B).

Quantitative analyses of individual lipid species (Fig. 4C-L) as well as lipid category data (Supplementary Fig. 6 demonstrated reproducible changes. These alterations were moderate in magnitude but consistent across experiments. Free cholesterol (FC) levels were noticeably elevated in KO TAM-like macrophages, but not in KO M0 cells (Fig. 4C, D). Within the phospholipid classes, phosphatidylethanolamine (PE) species containing long-chain polyunsaturated fatty acids were consistently reduced in IGF2BP2-deficient macrophages (Fig. 4E-H). Species likely to contain arachidonic acid (e.g., PE 38:4, PE 40:4, PE 40:5) and docosahexaenoic acid (e.g., PE 40:6) were particularly affected. When grouped by the number of double bonds, PE species with four or more double bonds (DB4-DB7) were significantly reduced in KO M0 macrophages Fig. 4F). TAM-like cells showed a similar trend (Fig. 4H). A similar pattern was observed for phosphatidylcholines (PCs), the most abundant class of membrane phospholipids. Highly unsaturated PC species, including PC 38:4, PC 38:5, PC 38:6, PC 40:5, and PC 40:6, were significantly decreased in both KO M0 and KO TAM-like macrophages (Fig. 4I-L). Grouped analysis further confirmed that DB4-DB6 PCs were consistently lower in IGF2BP2-deficient cells (Fig. 4J, L).

### Myeloid-Specific IGF2BP2 ΜΦ-KO Reduces Tumor Growth of Murine Subcutaneous Lewis Lung Carcinoma

**Figure 5:**
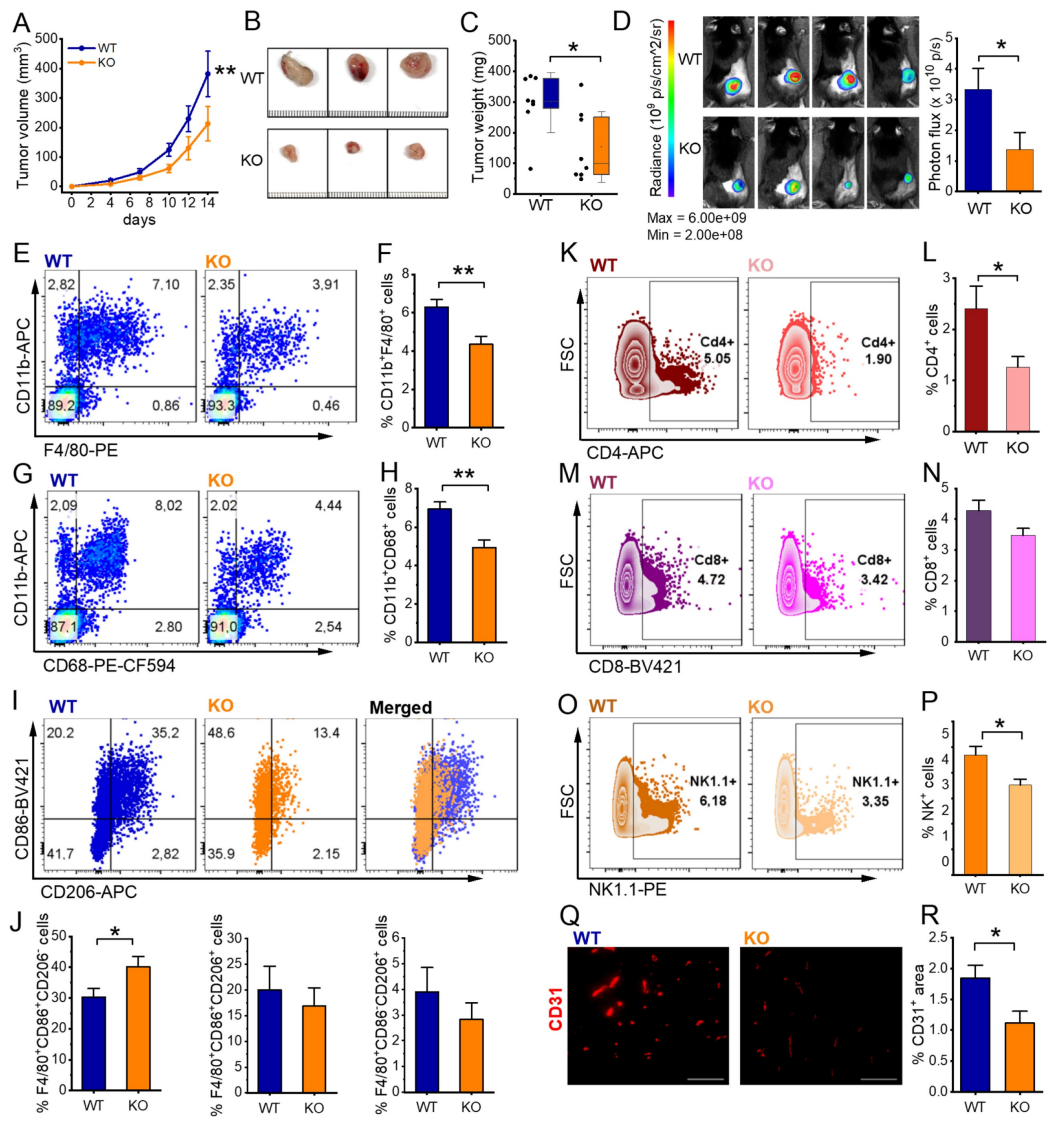
**IGF2BP2 ΜΦ-KO reduces tumor growth, alters immune cell infiltration, and impairs angiogenesis in LLC1 tumors.** Luciferase-expressing Lewis lung carcinoma (LLC1) cells were injected subcutaneously into WT and IGF2BP2 ΜΦ-KO mice. **(A)** Tumor volume. **(B)** Representative images of resected subcutaneous tumors, scale bar = 1 mm per line. **(C)** Comparison of tumor weights between WT and IGF2BP2 ΜΦ-KO mice. **(D)** Bioluminescence imaging of luciferase-expressing LLC1 cells following luciferin injection, with photon flux intensity quantified on day 14. **(E-J)** For flow cytometric analysis, tumors were collected and dissociated on day 14. **(E, G,** left panel**)** Representative dot plots showing TAMs, gated as CD11b⁺F4/80⁺ or CD11b⁺CD68⁺ populations **(F, H**, right panels**)** TAM quantification. **(I)** TAM polarization. Representative F4/80⁺-gated plots showing CD86 vs. CD206 for WT and KO, plus a merged/overlay panel (WT vs. KO) to illustrate population shifts.**(J)** Quantification of TAM subsets within F4/80⁺: CD86⁺CD206⁻ (M1-like), CD86⁺CD206⁺ (mixed), and CD86⁻CD206⁺ (M2-like), expressed as % of F4/80⁺ TAMs per tumor. **(K, M, O)** Representative FSC-A versus CD4, CD8, or NK1.1 plots of viable singlets with a right-hand rectangular gate delineating CD4⁺, CD8⁺, or NK1.1⁺ cell populations. **(L, N, P)** Quantification of CD4⁺, CD8⁺, and NK1.1⁺ cells, expressed as % of viable singlets per tumor.** (Q)** Vascularization was quantified as the CD31-positive area fraction. For each field (entire cropped image area), a global threshold was applied to generate a binary red mask, and the %CD31⁺ area was computed as 100 × (area of threshold-positive pixels / total area of the cropped field). Images were acquired with identical exposure settings. Scale bar = 100 µm. ((A-J) n = 8 mice per group, (Q) n = 8 WT, n = 7 ΜΦ-KO mice per group, from two independent experiments). Statistical significance was determined using two-way ANOVA for multiple time points (A) and Student's *t*-test for comparisons in (C-R) (*p < 0.05; **p < 0.01; ***p < 0.001).

To further investigate the role of IGF2BP2 in TAMs and its impact on myeloid cells within the tumor microenvironment (TME) and tumor progression, we used an LLC1 tumor model. We injected luciferase-expressing LLC1 cells subcutaneously into WT and IGF2BP2 ΜΦ-KO mice. As shown in Figure 5A-C, IGF2BP2 ΜΦ-KO mice developed significantly smaller tumors with reduced volume and weight compared to WT controls. Bioluminescence imaging on day 14 confirmed a lower tumor burden in ΜΦ-KO mice, as indicated by reduced radiance and photon flux quantification (Fig. 5D).

Flow cytometric analysis was performed to characterize the immune cell composition within the tumors. IGF2BP2 ΜΦ-KO mice exhibited a significant reduction in TAMs, defined as CD11b⁺F4/80⁺ or CD11b⁺CD68⁺ (Figure 5E-H). Given the well-established pro-tumorigenic role of TAMs 51, this reduction may contribute to the observed decrease in tumor growth. Further phenotypic analysis of TAMs using the CD86 and CD206 markers revealed a modest but significant increase in the CD86⁺CD206⁻ population in ΜΦ-KO mice, suggesting a shift toward an M1-like (classically activated) phenotype (Fig. 5I, J).

Beyond macrophages, myeloid-specific IGF2BP2 deficiency also affected other immune populations. Notably, the proportions of CD4⁺ and CD8⁺ leukocyte populations, consistent with T lymphocytes, were significantly reduced in ΜΦ-KO compared to WT tumors (Fig. 5K-N). Additionally, NK1.1⁺ natural killer (NK) cells were markedly reduced in ΜΦ-KO tumors (Figure 5O, P).

To evaluate tumor vascularization, we performed CD31 immunofluorescence staining. ΜΦ-KO tumors exhibited a significantly lower CD31-positive endothelial cell area compared to WT, indicating impaired angiogenesis (Fig. 5Q, R). Consistently, gene expression analysis showed a slight reduction in *Fgf2***,** *Hif1a***,** and *Mmp2*, while *Mmp9* expression was significantly decreased in IGF2BP2 ΜΦ-KO tumors Supplementary Fig. 7.

### Impaired Cell Migration in IGF2BP2 ΜΦ-KO Macrophages *in Vitro* and *in Vivo*

Tumor progression is closely linked to immune cell recruitment and migration 52. As shown above (Fig. 4), IGF2BP2-deficient macrophages exhibited substantial alterations in membrane lipid composition, including increased free cholesterol and decreased polyunsaturated phospholipids, both of which are known to impair membrane fluidity and potentially impede cellular motility. Given these biophysical changes and the reduced number of immune cells observed in IGF2BP2 ΜΦ KO tumors, we next investigated whether IGF2BP2 deletion directly affects macrophage migration.

**Figure 6:**
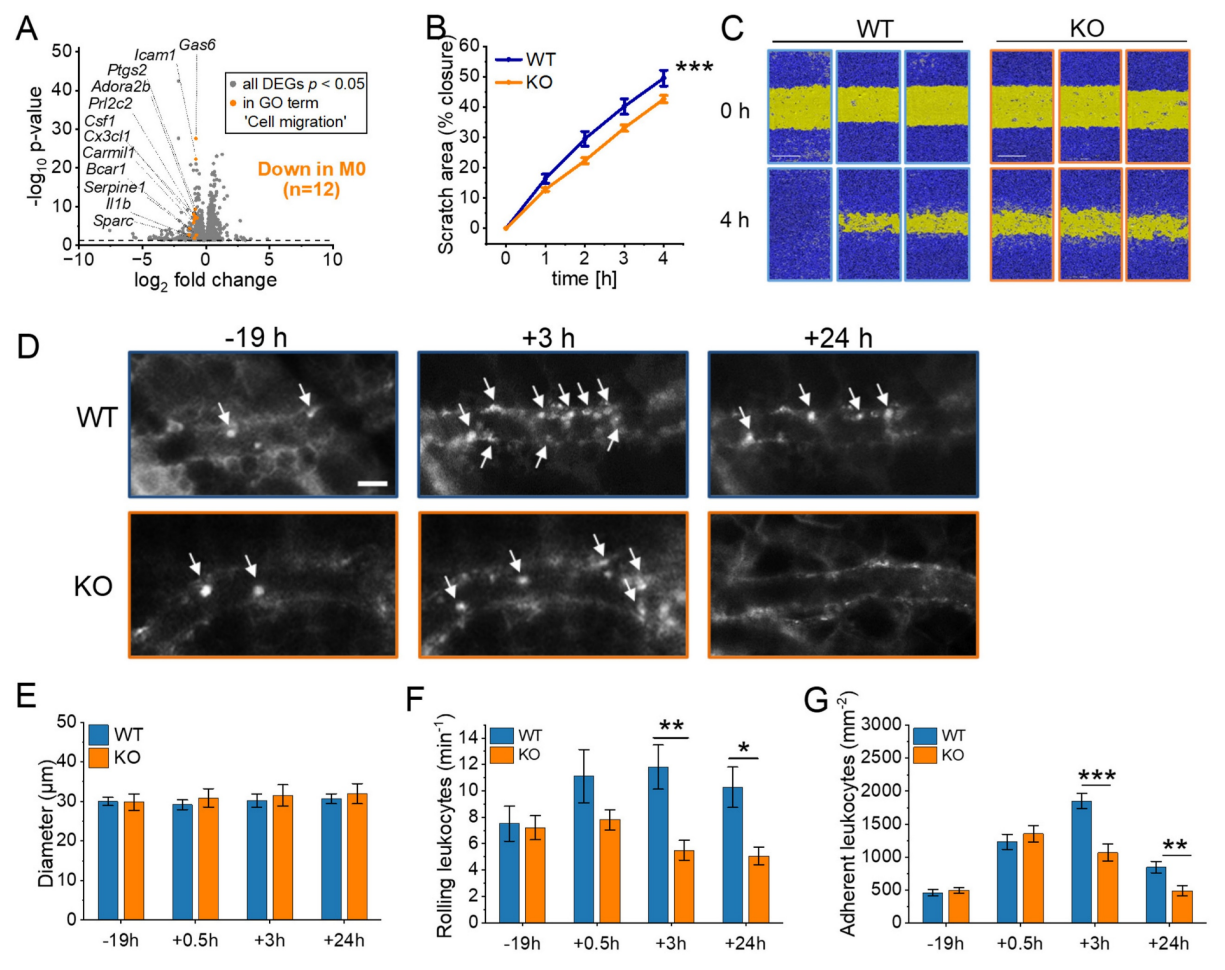
** IGF2BP2 deficiency impairs macrophage migration and reduces leukocyte recruitment *in vivo*. (A)** Volcano plots of DEGs (shown in gray), with significantly downregulated genes in the GO term “cell migration” (GO:0030335) highlighted in orange for M0 macrophages (n = 3 mice per group). **(B, C)** Live-cell microscopy-based analysis of cell migration. Following scratch, scratch area coverage (%) of WT and IGF2BP2 KO BMMs was monitored in an IncuCyte^®^ S3 system over 4 hours. Representative images are shown; scale bar = 400 µm (n = 7 mice per group, triplicates). **(D)** Intravital fluorescence microscopic images of postcapillary venules in the dorsal skinfold chamber of WT and IGF2BP2 ΜΦ-KO mice 19 h before and 3 and 24 h after LPS treatment. Green-light epi-illumination with staining of leukocytes (arrows) by rhodamine 6G. Scale bar = 30 μm. Quantification of **(E)** venous and arteriolar diameters**, (F)** rolling leukocytes, **(G)** adherent leukocytes in dorsal skinfold chambers of WT and IGF2BP2 ΜΦ-KO mice (n = 8 mice per group) that were exposed to LPS for 0.5 hours. WT and IGF2BP2 ΜΦ-KO animals were analyzed at corresponding time points. Measurements were obtained using intravital fluorescence microscopy and computer-assisted image analysis. Data are presented as mean ± SEM. Statistical significance was determined using two-way ANOVA for multiple time points (B) or Student's *t*-test for individual time points (E-G) (*p < 0.05; **p < 0.01; ***p < 0.001).

RNA-Seq revealed a significant downregulation of genes associated with cell motility, migration, and movement of cellular components in cluster 1 in IGF2BP2 KO macrophages (Fig. 1I, J). Specifically, 12 genes within the GO term "cell migration" were significantly reduced in M0 macrophages (Fig. 6A). To assess the functional consequences of these transcriptional changes, we performed a scratch assay *in vitro*, which demonstrated moderately reduced migratory capacity in IGF2BP2 KO macrophages compared to WT controls (Fig. 6B, C).

To examine leukocyte migration *in vivo*, we used the dorsal skinfold chamber model, which allows real-time visualization of leukocyte-endothelial interactions within striated muscle tissue. Migration was stimulated *via* topical LPS application. Previous experiments confirmed that WT and IGF2BP2 KO macrophages exhibited a comparable pro-inflammatory response to LPS, including similar cytokine mRNA expression, TNF secretion, and activation of ERK and NF-κB signaling pathways Supplementary Fig. 8.

Using intravital fluorescence microscopy, we analyzed key leukocyte migration processes, including rolling, adherence, and transmigration. To investigate the impact of IGF2BP2 depletion on myeloid cell migration, we utilized the LysMCre system, which induces IGF2BP2 deletion primarily in macrophages, neutrophils, and monocytes. This enabled us to assess the migration dynamics of these cell populations in real-time using the dorsal skinfold chamber model (Fig. 6D-G, Supplementary Fig. 9). The specificity of the LysMCre system for myeloid cells was previously validated 53.

As expected, venous and arteriolar diameters remained comparable between WT and IGF2BP2 ΜΦ-KO mice (Fig. 6E, Supplementary Fig. 9A). However, the IGF2BP2 ΜΦ-KO mice showed a significantly reduced number of rolling and adherent leukocytes in venules at 3 and 24 hours after exposure to LPS (Fig. 6F, 6G). In addition, the number of adherent leukocytes in arterioles was significantly reduced in the KO group at 3 and 24 hours Supplementary Fig. 9C).

## Discussion

Our study highlights the critical role of IGF2BP2 in regulating macrophage metabolism, polarization, and migration, with specific implications for TAMs and tumor progression. Using myeloid-specific IGF2BP2 ΜΦ-KO mice, we demonstrate that IGF2BP2 deficiency drives a metabolic shift favoring glycolysis over OXPHOS, impairs macrophage migration, and alters TAM polarization, leading to reduced tumor growth.

### IGF2BP2 as a Regulator of Macrophage Metabolism

Macrophages exhibit remarkable metabolic plasticity, adapting their energy pathways to environmental cues. Pro-inflammatory M1 macrophages rely on glycolysis and increased glucose uptake, whereas anti-inflammatory M2 macrophages depend on fatty acid oxidation (FAO) and OXPHOS for energy (54.

IGF2BP2 plays a critical role in metabolic transitions, with its KO in macrophages leading to enhanced glycolysis (increased ECAR) and impaired mitochondrial respiration (decreased OCR). KO cells display reduced mitochondrial membrane potential, indicating mitochondrial dysfunction, alongside lower mitochondrial ROS levels, suggesting decreased electron flow through the ETC. This aligns with previous findings showing that a reduction in mitochondrial membrane potential increases activation of the mitochondrial permeability transition pore, which inhibits oxidative phosphorylation, leading to lower mitochondrial ROS production and disrupted electron flow 55. Despite these functional impairments, the mitochondrial DNA content remained stable, with only a slight reduction in mitochondrial mass, indicating that IGF2BP2 primarily regulates mitochondrial activity rather than biogenesis. Interestingly, while our data demonstrate that IGF2BP2 deficiency in macrophages enhances glycolysis, several studies in tumor cells have shown the opposite effect: IGF2BP2 promotes glycolysis by stabilizing transcripts such as *ALDOA*, *GLUT1*, *HK2*, or the lncRNA *DANCR* 9,56-58. More recently, IGF2BP2 was identified as a critical factor in PLK1-overexpressing tumors, where its loss led to a marked reduction in PLK1 expression. This disruption further impaired mitochondrial metabolism and suppressed tumor cell proliferation and survival 59. This apparent discrepancy highlights the strong context- and cell type-dependence of IGF2BP2 function. In tumor cells, IGF2BP2 acts mainly by stabilizing glycolytic mRNAs, thereby directly promoting glycolysis. In macrophages, however, our findings suggest a different mechanism: IGF2BP2 deletion primarily impairs mitochondrial respiration, which in turn leads to a compensatory metabolic shift towards glycolysis. This dual role underlines the importance of considering cellular context when interpreting IGF2BP2's metabolic functions, particularly within the tumor microenvironment.

### IGF2BP2 Modulates TAM Polarization and the Tumor Microenvironment

Our study further revealed that IGF2BP2 plays a role in macrophage polarization. IGF2BP2 expression was elevated in TAM-like macrophages, and its deletion led to a reduction in selected TAM-associated genes, including *Mrc1*, *Mmp2*, and *Il10,* and a glycolytic shift. TAMs facilitate tumor progression by secreting cytokines that enhance tumor cell proliferation and suppress immune responses 60. Myeloid-specific IGF2BP2 deletion significantly reduced tumor growth in the LLC1 model, with ΜΦ-KO mice exhibiting smaller tumors, reduced tumor burden and TAM infiltration, and an altered macrophage phenotype. However, ΜΦ-KO tumors exhibited a reduction in CD4^+^ and CD8^+^ cell populations, consistent with T lymphocytes, as well as NK cells, indicating that IGF2BP2 deletion impacts not only macrophages but also the broader immune landscape. A likely explanation for reduced T and NK cell infiltration in IGF2BP2 ΜΦ-KO tumors is the altered recruitment capacity of TAMs. TAMs contribute to T cell recruitment *via* chemokines, such as CCL5, CCL2, and CXCL9/10 61. Thus, a decrease in TAM numbers, regardless of polarization, may reduce chemokine production and lower lymphocyte recruitment.

These findings align with previous studies highlighting the role of IGF2BP2 in macrophage polarization. Our prior research 20 showed that IGF2BP2 expression is elevated in M2 macrophages and chronic inflammation but downregulated in acute inflammatory responses, indicating a context-dependent role in macrophage activation.

Consistent with this, IGF2BP2 has been shown to promote an M2 phenotype in various contexts. Elevated IGF2BP2 expression in cancer cells modulates extracellular vesicle secretion, driving macrophages toward a TAM-like phenotype and promoting tumor progression 62. Similarly, IGF2BP2 stabilizes *NRP1* mRNA, facilitating M2 macrophage polarization and enhancing bladder cancer malignancy 8. In addition, high IGF2BP2 expression correlates with immunosuppressive M2-like macrophages, angiogenesis, and immune evasion in bladder cancer 63. Further supporting the role of IGF2BP2 in macrophage polarization, IGF2BP2 deletion enhances M1 polarization and promotes inflammation in colitis while reducing IL-4-induced M2 activation, suggesting that IGF2BP2 facilitates the M1-to-M2 transition 64.

Our findings also suggest species-specific differences in IGF2BP2 regulation. While IGF2BP2 expression was not induced in HMDMs after 24 hours of LPS treatment 20, it was upregulated in murine BMMs after 4 hours of LPS treatment without significantly altering the pro-inflammatory response. This contrasts with the findings of Wang et al., where IGF2BP2 ΜΦ-KO BMMs exhibited increased pro-inflammatory cytokine expression 64, and with studies in THP-1 cells, where IGF2BP2 acts as an m⁶A reader that binds to methylated TIM1 mRNA, enhancing its stability and thereby promoting M1 polarization and inflammation 65. Consistent with this, our data show that transcripts strongly downregulated in IGF2BP2 KO macrophages are significantly enriched in m^6^A motifs in their 3'UTRs, namely DRACH motifs 47, supporting a role for IGF2BP2 as an m^6^A-reader 66 stabilizing these mRNAs Supplementary file 4. These discrepancies highlight the complexity of IGF2BP2's role in macrophage polarization, influenced by differences in experimental models, polarization conditions, and species-specific responses. Murine macrophages generally exhibit a limited LPS response due to the rapid induction of negative feedback regulators 67. Moreover, human macrophages activated by LPS rely on oxidative phosphorylation rather than glycolysis for ATP generation 68, potentially contributing to the observed differences between murine and human macrophages.

### IGF2BP2 and Macrophage Migration

Macrophage migration is essential for tumor progression, as alterations in motility can significantly affect immune responses and cancer development 69. Our findings demonstrate that KO macrophages exhibit a markedly impaired migration capacity both *in vitro* and *in vivo*. In the dorsal skinfold chamber model, ΜΦ-KO mice had fewer rolling and adherent leukocytes at inflammation sites. RNA-Seq analysis revealed downregulation of migration-related genes in KO macrophages, which was further confirmed by a scratch assay showing reduced migration. Consistent with previous findings that IGF2BP2 activates RhoA/ROCK signaling 70, our Ingenuity Pathway Analysis (IPA) of IGF2BP2 KO macrophages revealed significant downregulation of pathways associated with actin-based cell motility, particularly those involving RhoA.

Beyond cytoskeletal signaling, membrane lipid composition emerged as a critical determinant of migratory capacity. Our lipidomic analyses revealed consistent but moderate alterations in IGF2BP2-deficient macrophages. Reductions in polyunsaturated phosphatidylethanolamine (PE) and phosphatidylcholine (PC) species, particularly those containing four or more DB arachidonic and docosahexaenoic acyl chains, were observed. We detected elevated free cholesterol (FC) concentrations in KO TAM-like macrophages. Such an increase in FC, characterized by a rigid and planar molecular structure, increases lipid packing and decreases membrane fluidity, negatively affecting dynamic cellular activities, including migration 71-73. Indeed, both PE and PC have been implicated in promoting cellular motility through increased membrane flexibility and curvature 71-73. Notably, a balanced phospholipid-to-free cholesterol (PL/FC) ratio has previously been reported to be essential for efficient macrophage phagocytosis 74. Thus, the observed lipid changes, notably the decreased polyunsaturated phospholipid species and increased cholesterol concentrations, collectively suggest impaired membrane fluidity and diminished functional plasticity of IGF2BP2-deficient macrophages. Nevertheless, these changes are unlikely to represent the sole mechanism underlying impaired migration; rather, they are one aspect of the broader metabolic reprogramming induced by IGF2BP2 loss.

These migratory defects were not attributable to altered acute inflammatory activation; upon LPS stimulation, both WT and KO BMMs mounted comparable pro-inflammatory responses, and no phenotypic changes were detected.

These findings are consistent with previous research suggesting that IGF2BP2 plays a pivotal role in immune cell recruitment to the TME, potentially through cytokines like CCL2 64. For instance, in oral squamous cell carcinoma, high IGF2BP2 expression was linked to reduced immune cell infiltration, suppressed immune responses, and fewer CD8^+^ T cells 75.

Another intriguing aspect of our findings relates to macrophage polarization, as metabolic reprogramming in KO TAM-like macrophages suggests a shift toward a more pro-inflammatory phenotype. While the exact relationship between macrophage phenotype and migration remains unclear, evidence suggests that morphology and motility are closely linked. For example, pro-inflammatory M1 macrophages typically exhibit more persistent and directed movement than anti-inflammatory M2 macrophages 76,77. This raises the possibility that IGF2BP2 may modulate migration not only through direct signaling pathways but also by influencing macrophage polarization and subsequent morphological changes.

This study has several limitations that should be considered when interpreting the results. The LysM-Cre model used to delete IGF2BP2 is not entirely specific for myeloid cells, raising the possibility of effects in other cell types 78. Furthermore, the number of biological replicates was relatively small, which reduces the statistical power to detect subtle effects. Some of the observed changes were moderate in magnitude and should be interpreted with caution. Finally, tumor experiments were performed exclusively in female mice, so sex-specific differences in the role of IGF2BP2 cannot be ruled out. Also, the TCM-based TAM-like model only partially reproduces the complex tumor-macrophage interactions occurring *in vivo*.

## Conclusions

Taken together, our study identifies IGF2BP2 as a key regulator of macrophage metabolism, polarization, and migration, with significant implications for TAM function and tumor progression. IGF2BP2 deletion induces a metabolic shift toward glycolysis, impairs TAM-like polarization, and disrupts macrophage migration, collectively contributing to reduced tumor growth. These findings suggest that therapeutic targeting of IGF2BP2 in myeloid cells may offer a novel approach to modulate the tumor microenvironment and inhibit cancer growth.

## Supplementary Material

Supplementary figures and table.

Supplementary file 1.

Supplementary file 2.

Supplementary file 3.

Supplementary file 4.

Supplementary file 5.

## Data Availability

Raw and processed RNA-sequencing data were deposited in the Gene Expression Omnibus (GEO) database under the accession code GSE292558. RNA-Seq data shown in Fig. 3 A and B were obtained from GEO under accessions GSE162669 and GSE162698.
