# Supplementary material for: IGF2BP2 Deficiency in Macrophages Impairs Migration, Reprograms Metabolism, and Limits Tumor Progression
Source: Int J Biol Sci. 2026 Feb 18;22(6):2754–73. doi: 10.7150/ijbs.122142 (PMC13050439; doi:10.7150/ijbs.122142)
Supplement: Supplementary file 1 — Supplementary figures and table. [file ijbsv22p2754s1.pdf]

Supplement

IGF2BP2 Deficiency in Macrophages Impairs Migration, Reprograms Metabolism, and Limits Tumor Progression

Hanna S. Schymik[1], Selina Wrublewsky[2,6,7], Marcus Höring[3], Gerhard Liebisch[3], Simon Both[1], Gilles Gasparoni[4], Caroline Bickelmann[2], Hanah Robertson[5], Charlotte Dahlem[1], Jörn Walter[4], Volkhard Helms[5], Matthias W. Laschke[2,6,7], Emmanuel Ampofo[2,6,7], Jessica Hoppstädter[1], Alexandra K. Kiemer[1,6,7*]

[1]Department of Pharmacy, Pharmaceutical Biology, Saarland University, Saarbrücken, Germany.

[2] Institute for Clinical & Experimental Surgery, Saarland University, Homburg, Germany.

[3] Institute of Clinical Chemistry and Laboratory Medicine, University Hospital Regensburg, Regensburg, Germany.

[4] Department of Genetics/Epigenetics, Saarland University, Saarbrücken, Germany.

[5] Center for Bioinformatics, Saarland University, Saarbrücken. Germany

[6] Center for Gender-Specific Biology and Medicine (CGBM), Saarland University, Homburg, Germany.

[7] PharmaScienceHub (PSH), Saarland University, Saarbrücken, Germany.

***Correspondence:**

Alexandra Kathrin Kiemer, Pharmaceutical Biology, Saarland University, D-66123 Saarbrücken, Germany. E-mail: pharm.bio.kiemer@uni-saarland.de

Supplemental Tables

Table 1: Primer Sequences

Gene	Accession number	Primer forward sequence	Primer reverse sequence
Acss2	NM_019811.3	TATGTGACCGGAGATGGCTGC	TCGTGTTCCACAAGTGCCGA
Aldh2	NM_001308450	GGACCTACCTAGCGGCCTTG	TGGTACTTGTCAGCCCAGCC
Aldoc	NM_001303423	CATCTGCCAGCAGAATGGGAT	GCTTGAGCAGAGTCCCTTCG
Bpgm1	NM_007563.5	GTCTGCAGCACGGCGTTAC	CAAGAACAGCAGTATATCTGCAAGG
Cxcl1	NM_008176.3	AACCGAAGTCATAGCCACACT	CCGTTACTTGGGGACACCTT
Fgf2	NM_008006.2	GCTCTACTGCAAGAACGGCG	TAACACACTTAGAAGCCAGCAGC
Gapdh	NM_001289726.2	TCGGTGTGAACGGATTTGGC	TGAAGGGGTCGTTGATGGCA
Gpi1	NM_008155.4	CCTGTCTACGAACACGGCCA	GCTGACCACAGCGAATAGCG
Hif1a	NM_001313919	TCAAGCAGCAGGAATTGGAACAT	TCATCCATTGACTGCCCCAGC
Hk2 (genomic DNA)	NC_000072.7	GCCAGCCTCTCCTG ATTTTAGTGT	GGGAACACAAAAGACCTCTTCTGG
Il1β	NM_008361.3	CCAAAAGATGAAGGGCTGCTT	GGAAGGTCCACGGGAAAGAC
Il6	NM_031168.2	AAGAAATGATGGATGCTACCAAACTG	GTACTCCAGAAGACCAGAGGAAATT
Igf2bp2	NM_183029.2	TTGGATGGGCTGTTGGCTGA	GTGACGTTGACAACGGCAGTT
Mito 16S	AP014886.1	CCGCAAGGGAAAGATGAAAGAC	TCGTTTGGTTTCGGGGTTTC
Mito Nd1	AP014886.1	CTAGCAGAAACAAACCGGGC	CCGGCTGCGTATTCTACGTT
Mmp2	NM_008610.2	CATCGCTGCACCATCGC	GCATGGTCTCGATGGTGTTC
Mmp9	NM_013599.3	GCCGACTTTTGTGGTCTTCC	TACAAGTATGCCTCTGCCAGC
Mrc1	NM_008625.2	TTCAGCTATTGGACGCGAGG	GAATCTGACACCCAGCGGAA
Nos2	NM_010927.3	CTTCCTGGACATTACGACCC	TACTCTGAGGGCTGACACAA
Pgk1	NM_008828.3	CAAGCTGGACGTGAAGGGGAA	AACAGCAGCCTTGATCCTTTGGT
Ppia	NM_008907.1	GCGTCTCCTTCGAGCTGTTT	CACCCTGGCACATGAATCCT
RNA18S	NR_003278.3	AGGTCTGTGATGCCCTTAGA	GAATGGGGTTCAACGGGTTA
Tgfb	NM_011577.1	ACCCTGCCCCTATATTTGGA	CGGGTTGTGTTGGTTGTAGAG
Tnf	NM_013693.2	CCATTCCTGAGTTCTGCAAAGG	AGGTAGGAAGGCCTGAGATCTTATC
Vegfa	NM_001025250.3	CCACCATGCCAAGTGGTCCC	ACCAGGGTCTCAATCGGACG

Supplemental Figures

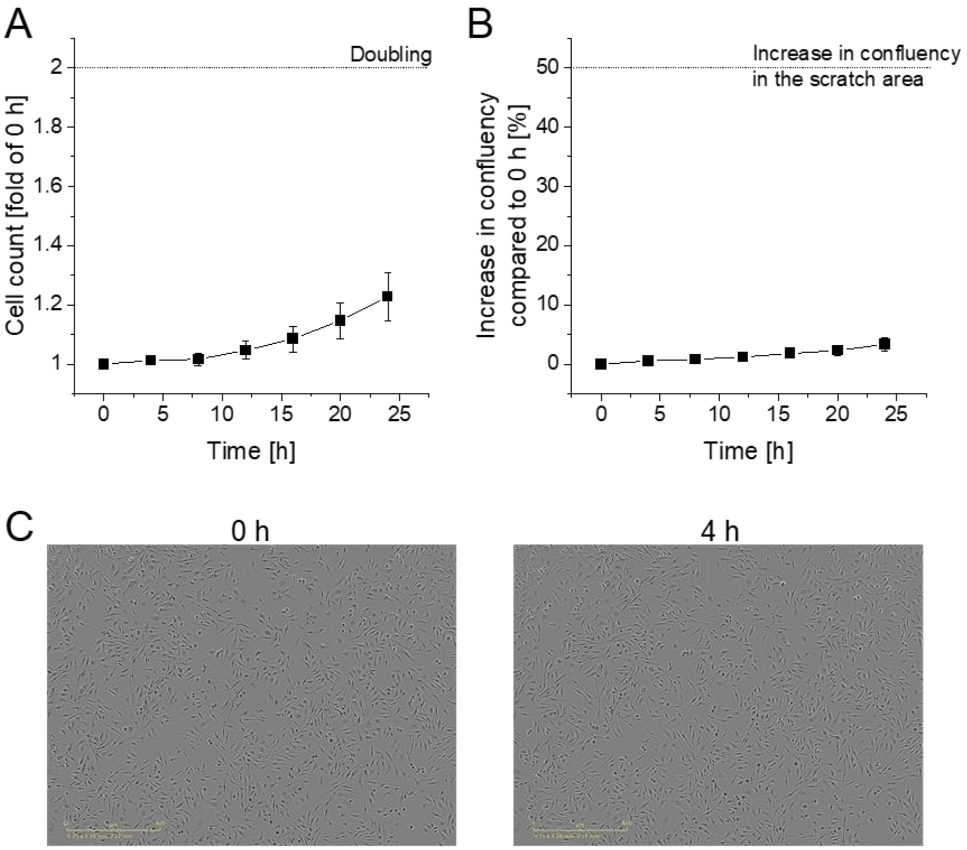

Supplemental Figure 1. Proliferation of bone marrow-derived macrophages under culture conditions used for migration assays (n=8). (A) Cell counts over 24 h, normalized to baseline at 0 h. BMMs showed only a 1.2-fold increase after 24 h. (B) Confluency increase measured by IncuCyte live-cell imaging. Confluency increased by 3.3 % after 24 h, corresponding to ~0.13 % per hour. (C) Representative phase-contrast images of BMM cultures at 0 h and 4 h, illustrating negligible changes in cell density over the assay period. Scale bar: 400 µm.

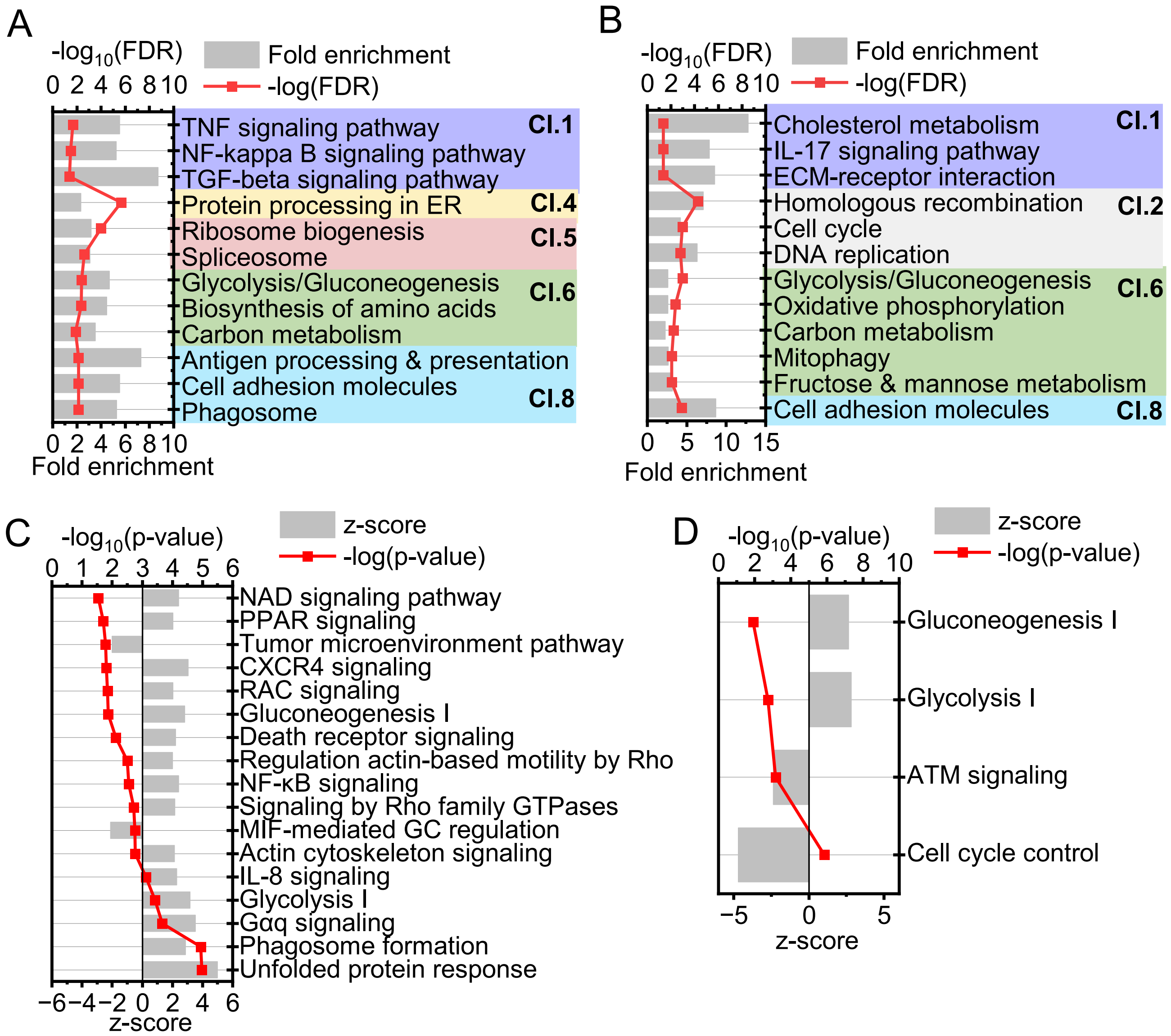

Supplemental Figure 2. Pathway Analysis in WT and IGF2BP2 KO Macrophages.
(A, B) Selected KEGG pathways enriched in clusters (Cl) identified as shown in Figure 1C and 1D. Pathway enrichment significance is shown by false discovery rate (FDR) values (fold change > 2, FDR < 0.05). **(C, D)** Ingenuity Pathway Analysis (IPA) of differentially expressed genes (DEGs; p < 0.05) in untreated **(A, C)** and IL-4-treated (8 h, 20 ng/ml) (B, D) BMMs (n = 3 mice per group) from WT and IGF2BP2 MΦ-KO mice n = 3 mice per group) (Supplementary file S5). Pathways are ranked by activation z-scores (z-score > 2, *p*-value < 0.05), indicating predicted pathway activation states.

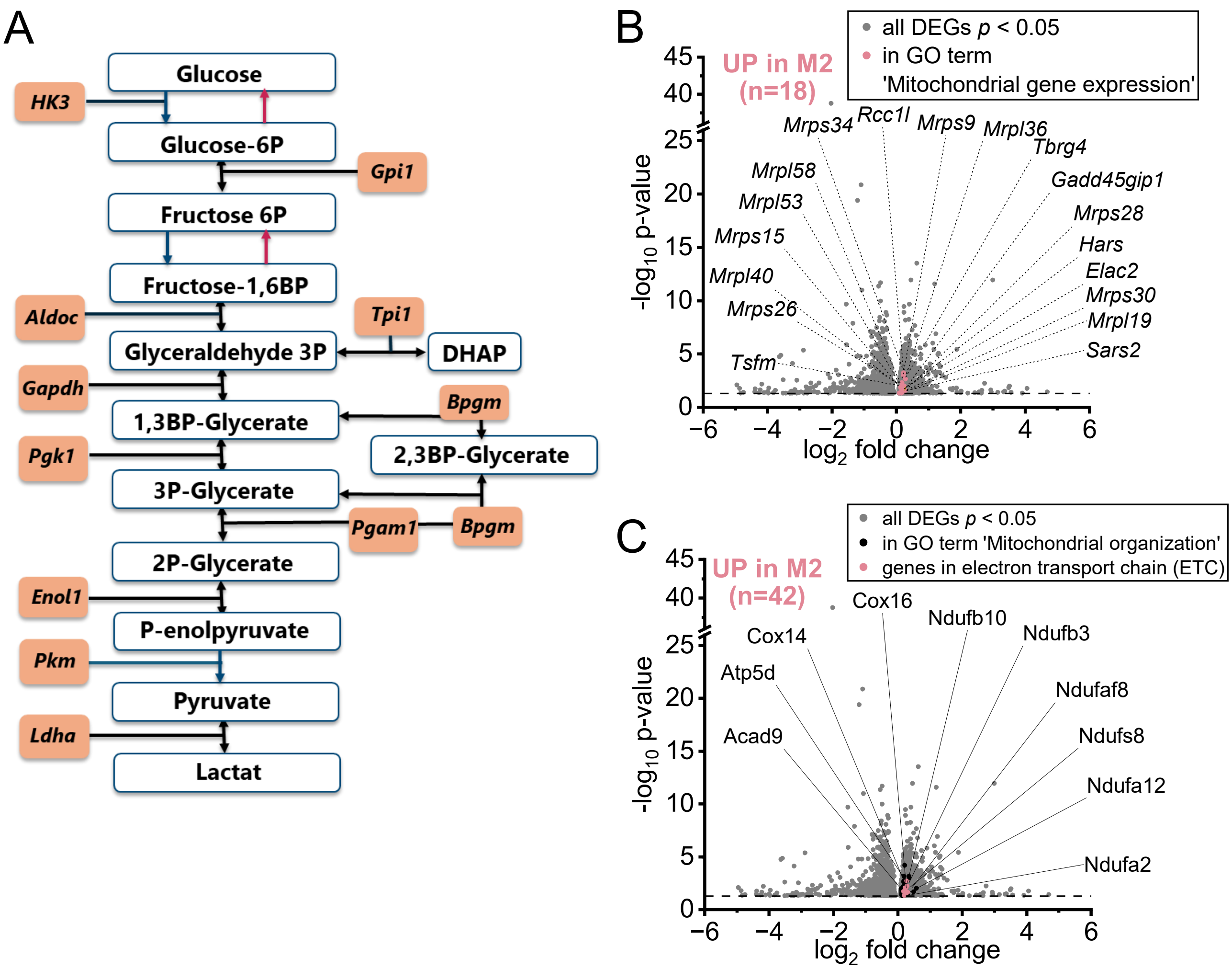

Supplemental Figure 3. Alterations in Glycolytic and Mitochondrial Pathways in IGF2BP2 KO Macrophages. (A) Schematic representation of the glycolytic pathway, highlighting genes (purple) that are significantly upregulated ($p < 0.05$) in IGF2BP2 KO M2-polarized macrophages in KEGG pathway "Glycolysis" **(B, C)** Volcano plots of DEGs (shown in gray) of KO M2 vs. WT M2 macrophages, with significantly upregulated genes in **(B)** the GO term "Mitochondrial gene expression" (GO:0140053) highlighted in purple, and **(C)** the GO term "Mitochondrial gene expression" (GO:0140053) shown in black, with a subset of genes from the electron transport chain (ETC) highlighted in purple (n = 3 mice per group).

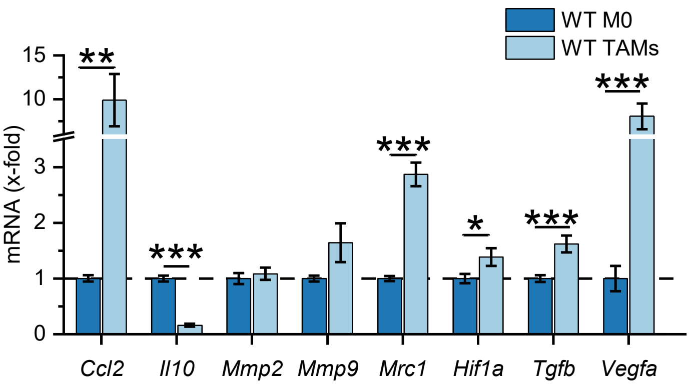

Supplemental Figure 4. Elevated TAM Marker Expression in LLC1 TAM-like Macrophages. BMMs were cultured in standard medium (M0) or TCM from LLC1 cells (TAM-like) for 8 hours. mRNA expression was measured by qPCR and normalized to *Ppia*. Expression of TAM-like macrophage markers is shown as a fold change of WT M0 (n = 6 mice per group, duplicates). Data are presented as mean ± SEM. Statistical significance was determined by Student's t-test (*$p < 0.05$; **$p < 0.01$; ***$p < 0.001$).

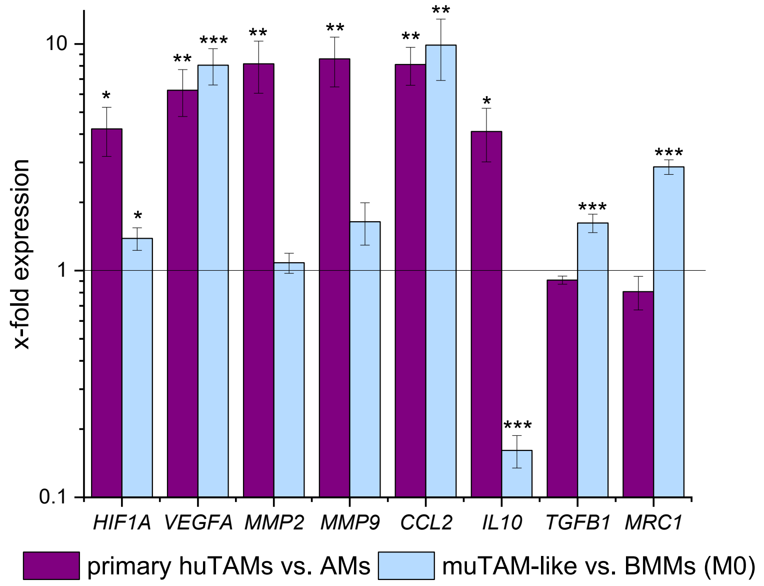

Supplemental Figure 5. Comparison of TAM-associated gene expression in murine and human TAM(-like) macrophages. mRNA expression of selected TAM-associated genes in murine BMMs cultured in standard medium (M0) or tumor-conditioned medium from LLC1 cells (TAM-like; n = 6 mice per group, duplicates) and in human tumor-associated macrophages (TAMs) versus matched alveolar macrophages (AMs) from GEO dataset GSE162669 (n = 3 donors per group, triplicates). Expression values are shown as x-fold change relative to M0 (for murine data) or AMs (for human data). Data are presented as mean ± SEM. Statistical significance was determined by Student's t-test (*p < 0.05; **p < 0.01; ***p < 0.001).

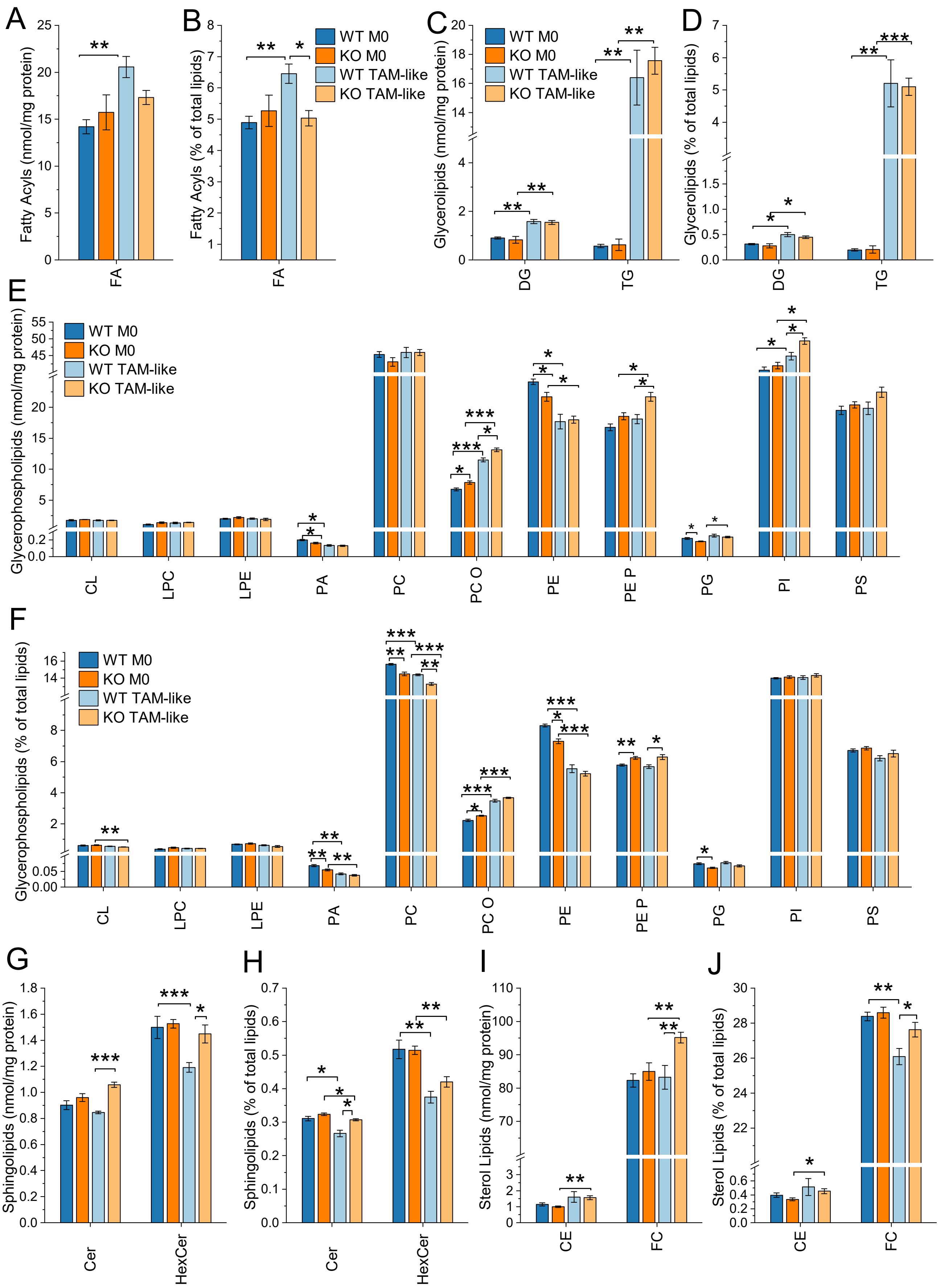
A
Fatty Acyls (nmol/mg protein)
FA
B
Fatty Acyls (% of total lipids)
FA
WT M0
KO M0
WT TAM-like
KO TAM-like
C
Glycerolipids (nmol/mg protein)
DG
TG
D
Glycerolipids (% of total lipids)
DG
TG
E
Glycerophospholipids (nmol/mg protein)
CL
LPC
LPE
PA
PC
PC O
PE
PE P
PG
PI
PS
F
Glycerophospholipids (% of total lipids)
G
Sphingolipids (nmol/mg protein)
Cer
HexCer
H
Sphingolipids (% of total lipids)
I
Sterol Lipids (nmol/mg protein)
CE
FC
J
Sterol Lipids (% of total lipids)

63

Supplemental Figure 6. Lipid Classes in WT and IGF2BP2 KO Macrophages (M0 and TAM-like macrophages). BMMs were cultured either in standard medium (M0) or in TCM from LLC1 cells for 24 hours to induce TAM-like polarization. Lipid concentrations in WT and IGF2BP2 KO M0 and TAM-like macrophages were analyzed by mass spectrometry. Data are displayed as **(A, C, E, G, I)** absolute concentrations (nmol/mg protein) and **(B, D, F, H, J)** relative abundance, expressed as a percentage of total detected lipids. Lipid categories: **(A-B)** Fatty acyls, **(C-D)** Glycerolipids, **(E-F)** Glycerophospholipids, **(G-H)** Sphingolipids, **(I-J**) Sterol lipids. Data are presented as mean ± SEM (n = 4 mice per group). Statistical significance was determined by ANOVA with Bonferroni's post hoc test (*$p < 0.05$; **$p < 0.01$; ***$p < 0.001$).

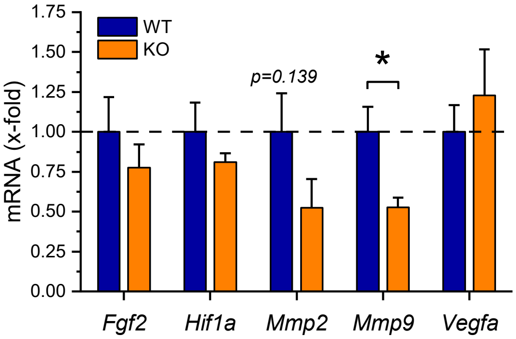

Supplemental Figure 7. Expression of Angiogenesis-related Genes in IGF2BP2 MΦ-KO Tumors. RNA was isolated from tumor tissue and mRNA expression levels of *Fgf2*, *Hif1a*, *Mmp2*, *Mmp9*, and *Vegfa* were normalized to *Ppia* and expressed as fold change relative to WT tumor tissue. Data are presented as mean ± SEM (n = 8 WT, n = 6 KO). Statistical analysis was performed using Student's t-test (*$p < 0.05$; **$p < 0.01$; ***$p < 0.001$).

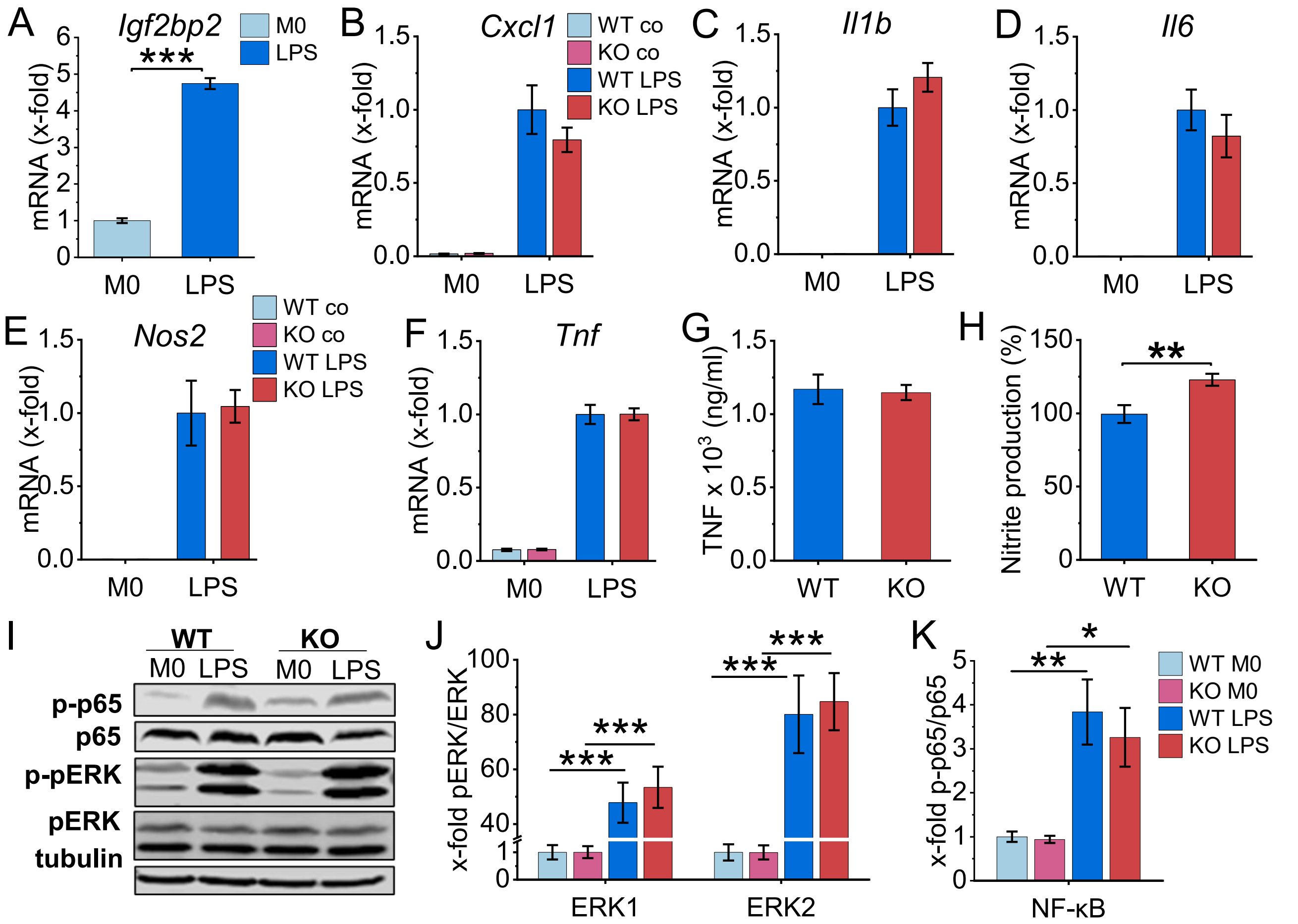

Supplemental Figure 8. Regulation and Role of IGF2BP2 in LPS-activated BMMs (A-G) BMMs were treated with 100 ng/ml LPS for 4 hours. **(A, B)** mRNA expression data were normalized to *Ppia* and expressed as x-fold of untreated WT control. A: *Igf2bp2* expression (n = 8 mice per group, duplicates). **(B-F)** Expression of *Cxcl1*, *Il1b*, *Il6*, *Nos2*, and *Tnf* (n = 5 mice per group, duplicates). **(G)** TNF secretion was measured by ELISA in LPS-treated BMMs (n = 8 mice per group, duplicates). **(H)** The relative nitrite production in LPS/IFN-γ (100 ng/ml; 25 ng/ml; 24 h) treated BMMs was measured by Griess assay, with data normalized to WT (set to 100%) (n = 8 mice per group, triplicates). **(I)** ERK and NF-κB activation in WT and IGF2BP2 KO BMMs were assessed by Western blotting after 15 minutes of LPS (100 ng/ml) treatment. **(J, K)** Phosphorylated ERK (pERK) and phosphorylated p65 (pp65) signal intensities were quantified and normalized to their respective total (unphosphorylated) protein levels. Tubulin served as a loading control (n = 5 mice per group, duplicates). Data are presented as means ± SEM. Statistical analysis was performed using ANOVA with Bonferroni's post hoc test (J,K) or Student's *t*-test (A, H) ($p < 0.05$; *$p < 0.01$; **$p < 0.001$).

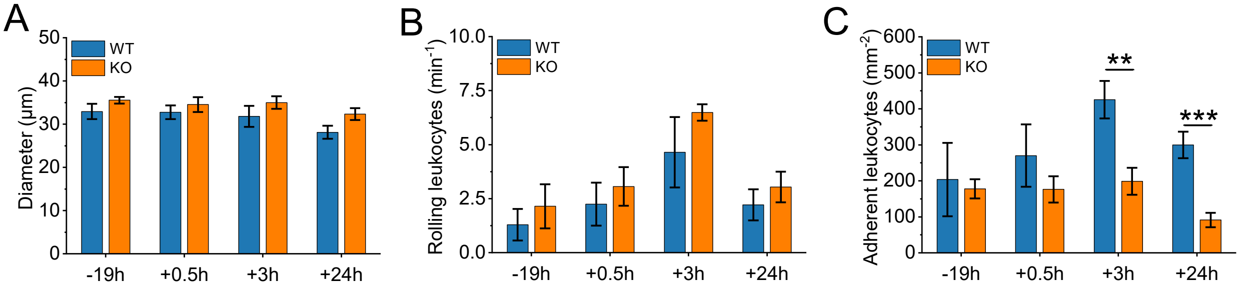

Supplemental Figure 9. IGF2BP2 Modulates Leukocyte Adherence in Arterioles. Intravital fluorescence microscopy of arterioles was used to assess microcirculatory changes. Quantification includes **(A)** arteriolar diameter (µM), **(B)** rolling leukocytes, and **(C)** adherent leukocytes in dorsal skinfold chambers of WT and IGF2BP2 MΦ-KO mice (n = 8 mice per group). Mice were exposed to LPS treatment for 0.5 hours, and WT and IGF2BP2 MΦ-KO animals were analyzed simultaneously. Measurements were obtained using intravital fluorescence microscopy and computer-assisted image analysis. Data are presented as mean ± SEM, with statistical significance determined using Student's t-test for individual time points (*p < 0.05; **p < 0.01; ***p < 0.001).
